# Tuning the Testicular Microenvironment for Enhancing Human Sertoli Cells Maturation and Functionality In Vitro

**DOI:** 10.1002/adhm.202505848

**Published:** 2026-04-24

**Authors:** Annachiara Scalzone, Giorgia Imparato, Chiara Ausilio, Valentina Mollo, Paolo Antonio Netti

**Affiliations:** ^1^ Center For Advanced Biomaterials For Healthcare (CABHC) Istituto Italiano di Tecnologia Naples Italy; ^2^ Dipartimento Di Ingegneria Chimica dei Materiali e della Produzione Industriale (DICMAPI) Università degli Studi di Napoli Federico II Naples Italy

**Keywords:** human Sertoli cells, in vitro tissue models, microenvironment, testis

## Abstract

Spermatogenesis relies on the highly specialized interaction between germ cells and a supportive somatic niche, yet replicating this environment in vitro remains a major challenge. Primary human Sertoli cells (hSCs), key architects of this niche, often lose their phenotype under conventional culture conditions, limiting the establishment of a stable blood‐testis barrier (BTB)‐associated phenotype and their ability to support germ cells. Here, we present a structurally organized, human ECM‐derived connective tissue equivalent (CTE) designed to support long‐term maintenance and organization of hSCs in vitro. The CTE, generated from fibroblast‐secreted matrix enriched in laminin, fibronectin, and collagen IV, reproduces key biochemical and physical features of a supportive microenvironment. hSCs introduced into the CTE as single cells or pre‐formed spheroids were evaluated for survival, structural organization, phenotypic stability, and ECM remodeling. Both configurations supported progressive expression and organization of BTB‐associated proteins (ZO‐1, OCLDN) together with upregulation of Sertoli cell‐associated markers, including SOX9 and ABP, with the spheroid‐based model showing improved structural cohesion, integration within the construct, and more evident junctional organization over time. Overall, this bioactive human‐derived platform supports long‐term maintenance of hSC phenotype and barrier‐associated features in vitro, providing a promising basis for future human testis models and co‐culture studies.

## Graphical abstract

An in vitro testicular model is developed by generating connective tissue equivalents from human dermal fibroblast‐derived microtissues and coupling them with human Sertoli cells or human Sertoli cell spheroids. This engineered microenvironment supports Sertoli cell maturation and functionality, providing a promising platform for studying human testicular physiology in vitro.

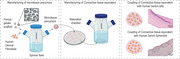

## 1 Introduction

Spermatogenesis is a highly coordinated cellular process involving the proliferation and differentiation of both germ and somatic cells, including Leydig and the Sertoli cells (SCs), within seminiferous tubules (STs), to generate high number of spermatozoa which constitute the fundamental cellular framework supporting male reproductive capacity. SCs orchestrate spermatogenesis by providing structural support, metabolic substrates, and regulatory cues within the specialized testicular niche, known as the seminiferous epithelium [1]. A hallmark of this microenvironment is the blood‐testis barrier (BTB), established by tight junctions between adjacent SCs, which dynamically remodel to regulate germ cell differentiation, facilitate their migration toward the lumen during spermatogenesis and protect them from immune surveillance. The extracellular matrix (ECM) within the interstitial space and basal lamina is a key component of this niche. Beyond its structural and mechanical function, the ECM also acts as a reservoir for biochemical cues, including growth factors and cytokines, that regulate SCs polarity, adhesion and BTB formation [2]. Key components, such as laminin, fibronectin, and collagen IV, are particularly relevant [3].

ECM disorganization, as observed in fibrotic testes from transfeminine individuals undergoing estrogen therapy, is associated with impaired Sertoli‐germ cell interactions and collagen accumulation that disrupts niche function [4]. While it is recognized that ECM's biochemical and mechanical properties, including stiffness, architecture, and topography, are critical modulators of cellular phenotype and function, the mechanisms through which ECM cues regulate SCs maturation and long‐term functionality remain poorly understood [5]. Recent reconstructions of seminiferous tubule (STs)‐like structures in 3D using mouse cells and decellularized ECM highlight the instructive role of testis‐derived matrix in promoting Sertoli cell polarization and tubulogenesis [6].

This knowledge gap is critical, because primary human SCs (hSCs) often lose their phenotype or fail to mature when cultured in vitro, significantly limiting the utility of existing models. During embryonic testis development, SCs and germ cells self‐organize into cord‐like structures, precursors to the STs, via mesenchymal‐epithelial interactions and matrix remodeling. Replicating such architecture in vitro remains technically challenging but is crucial for restoring SC function. Most current models such as 2D monolayers [7], Transwell‐based 2.5D platforms [8], and 3D organoids [5, 9], fail to maintain SCs functional stability over time and lack the environmental complexity needed for their differentiation, yet not fully capture the complexity of the native testicular niche [10]. As a result, they are often unable to drive full SCs maturation or recapitulate BTB dynamics. To overcome these limitations, several in vitro models have been developed to investigate SCs physiology and the formation of the BTB. Recent advances in testicular organoid technology have demonstrated promising capabilities in mimicking testicular architecture and function. For instance, Pendergraft et al. developed a 3D human testis organoid system incorporating SC to evaluate gonadotoxic potential and germ cell differentiation, successfully recapitulated BTB formation and SC functionality. However, the use of immortalized cell lines limits physiological relevance [11]. Similarly, Alves‐Lopes et al. showed enhanced SC maturation in 3D culture, though their rat‐derived system raises concerns about human applicability [5]. A common limitation among these models is their reliance on non‐bioactive simplified ECM substrates, such as methylcellulose or agar, which lack the necessary biophysical and biochemical complexity required for SC adhesion, differentiation, and proper function [12, 13].

Supporting evidence from Hadley et al. demonstrated increased androgen‐binding protein expression in SCs cultured on native ECM [14], while Zhang et al. showed that ECM‐mediated spatial organization drives ST‐like tubulogenesis in mouse cells [6]. To address these limitations, several advanced biomaterials have been investigated: collagen hydrogels support SC adhesion but lack mechanical stability; Matrigel enhances polarity but is undefined and tumor‐derived; decellularized testis ECM provides native cues but suffers from batch variability; chitosan scaffolds support SC function but require functionalization; and alginate, though biocompatible, is non‐bioactive unless modified [6, 15, 16, 17, 18, 19, 20]. Taken together, this evidence supports the notion that only models integrating physiologically relevant ECM components with appropriate biochemical and biomechanical cues, can sustain SC function and support their aggregation and partial structural organization within the ECM. Such features are essential not only for maintaining SCs phenotype, but also for enabling the reconstruction of the testicular niche [21].

Building on this premise, we investigate whether a human ECM‐derived microenvironment can sustain the long‐term maintenance, structural organization, and junctional features of primary hSCs in vitro. To this end, we introduce a native‐like connective tissue equivalent (CTE), a human‐derived, structurally organized ECM platform enriched in key testicular components (laminin, fibronectin, collagen IV). Unlike inert scaffolds or animal‐derived matrices, the CTE provides a bioactive microenvironment that supports hSC adhesion, structural integration, and ECM remodeling. When seeded with hSC spheroids, the system promotes enhanced cell–cell interactions and qualitatively improved spatial organization within the construct, together with a progressive organization of junction‐associated proteins. In this study, we specifically assess (i) the maintenance of hSC phenotype, via gene expression analysis; (ii) the organization of junction‐associated proteins linked to barrier‐related features, by immunofluorescence; (iii) the structural integration of hSCs within the ECM, and ECM remodeling within the construct, performing histological characterization. Within this framework, the model is intended to investigate how a human‐derived ECM microenvironment contributes to the organization and maintenance of primary hSCs in vitro, by supporting the preservation of key structural and junctional features that are typically lost in conventional in vitro systems.

## 2 Materials and Methods

### 2.1 hDF Culture

Human dermal fibroblasts (hDFs) were isolated from foreskin or breast skin biopsies as previously described [22, 23]. Cells were expanded in 150 cm^2^ flasks using Eagle's Basal Salt Solution Minimum Essential Medium (MEM) supplemented with 20% fetal bovine serum (Merck), 100 mg/mL L‐glutamine (Merck), 100 U/mL penicillin/streptomycin (Himedia, Einhausen, Germany), and 0.1 mm non‐essential amino acids (Euroclone, Italy). Cultures were maintained at 37°C in a humidified atmosphere with 5% CO_2_ and utilized at passage 7.

### 2.2 hSC Culture and Spheroids Formation

Human Sertoli cells (hSC, pubertal) (Cat. #4520, ScienCell) were cultured in Sertoli cell medium (Cat. #4521, ScienCell) according to the manufacturer's protocol. Prior to cell seeding, a T‐75 flask was coated with poly‐L‐lysine (PLL, 2 µg/cm^2^) by incubating 10 mL of sterile water with 15 µL of poly‐L‐lysine stock solution (10 mg/mL, Cat. #0413, ScienCell) at 37°C for at least 1 h or overnight. Following incubation, the flask was rinsed twice with sterile water, and 20 mL of complete medium was added. Cryopreserved hSCs were thawed by gently rotating the vial in a 37°C water bath until fully liquified. The cells were then resuspended in fresh medium and transferred to the coated flask. The culture was maintained at 37°C with 5% CO_2_, and after 16 h, the medium was replaced to remove residual DMSO and unattached cells. hSCs were expanded and passed up to passage 5, when these were used for experiments. hSC were studied in 2.5D systems to optimize the culture conditions.

For the formation of hSC spheroids, 60 000 cells were seeded into each well of a 96‐well round‐bottom ultra‐low attachment plate (Costar, Corning) and cultured for 3 days to facilitate the formation of compact spheroids. During this period, spheroids were maintained in growth medium under standard culture conditions.

### 2.3 hSC Culture Conditions in 2D Transwell System

hSC were cultured as reported in section 2.2. Transwell inserts (0.4 µm pore size, 0.33 cm^2^ surface area, PET, Corning) were coated with 100 µL of human fibronectin (6.6 µg/mL, Merck) diluted in PBS and incubated 40 min at 37°C, followed by an additional 10 min at RT. hSC (passage 5) were collected and seeded at a density of 5 × 10^5^ cells in 300 µL of medium in the apical chamber, while 900 µL of medium was added to the basolateral chamber of each 24‐well transwell insert. Three different conditions were exploited: (i) hSC in DMEM/F12 supplemented with 10% FBS, 1% P/S, 2% NEAA (hSC); (ii) hSC in DMEM/F12 supplemented with 10% FBS, 1% P/S, 2% NEAA, 100 ng/mL follicle‐stimulating hormone (FSH, cat. #HY‐P70237, MedChemExpress), and 10 µm Testosterone (cat. # 86500, Merck) hSC‐FSH‐T) [24, 25, 26]; (iii) hSC in DMEM/F12 supplemented with 10% FBS, 1% P/S, 2% NEAA, 100 ng/mL FSH, and 10 µm Testosterone (hSC‐FSH‐T) in Air‐Liquid interface conditions (hSC‐FSH‐T‐ALI). Cells were cultured under standard conditions in an incubator at 37°C with 5% CO_2_ up to 7 days in vitro (DIV), with media changes at DIV4. At DIV1, DIV4, and DIV7 transepithelial resistance (TEER) was measured at three different points per insert using a MilliCell Electrical Resistance System (ERS) epithelial volt‐ohmmeter (Millipore Corporation). Samples were depleted from medium and TEER measurements were performed in PBS. TEER values were normalized by subtracting the resistance of fibronectin‐coated control wells, in PBS, without cells.

### 2.4 Scanning Electron Microscopy (SEM)

Samples were fixed overnight at 4°C in 2.5% glutaraldehyde (Electron Microscopy Sciences, EMS) in 0.1 M sodium cacodylate Buffer (EMS). After washing in sodium cacodylate buffer, samples were post‐fixed in 1% osmium tetroxide (OsO_4_) aqueous solution (EMS) for 1 h at 4°C in the dark before dehydration. Dehydration was performed in an ascending series of ethanol (30%; 50%; 70%; 2% × 95%; 3% × 100%, Merck), with each step lasting 15 min on ice. The EM CPD 300 (Leica Microsystems) critical point dryer was used to completely dry the samples. Each sample was mounted on a 12 mm pin aluminum stub (Agar Scientific) with carbon tape and then sputtered with a 20 nm thickness of gold using the HR208 sputter coater (Cressington). Imaging was performed using a Field Emission Gun Scanning Electron Microscope (FESEM, Ultraplus_Zeiss) at 5 KV in Secondary Electrons (SE) mode, with magnification setting ranging from 1 to 50 kx.

### 2.5 Transmitted Electron Microscopy (TEM)

Samples were fixed in 4%PFA for 30 min, followed by fixation in 2.5% glutaraldehyde in 0.1 M sodium cacodylate buffer for 3 h at room temperature. After washing in sodium cacodylate buffer, the specimens were post‐fixed in 1% OsO_4_ and 1% potassium ferrocyanide (EMS) solution for 1 h at 4°C, in the dark. To enhance the plasma membrane, the samples were incubated for 3 min in 0.15% acid tannic aqueous solution and rinsed in distilled water at 4°C. Dehydration was performed in an ascending series of ethanol (30%; 50%; 70%; 2% × 95%; 3% × 100%, Merck) with each step lasting 15 min on ice before epoxy resin embedding. The samples were embedded overnight in a 1:1 mixture of absolute ethanol/low Viscosity resin (EMS) followed by embedding in absolute resin overnight. This was followed by three changes of resin, each lasting 2–3 h, before initiating polymerization in an oven at 70°C. Cross‐sections of 60 nm from each sample were obtained using an ultramicrotome (UC7/FC7, Leica), collected onto copper grids (Agar Scientific), and stained with a 1% uranyl solution. TEM imaging was performed using a Tecnai G2‐20 (FEI Company) at 120 kV, with a magnification range of 5–80 kx.

### 2.6 Focused Ion Beam Scanning Electron Microscope (FIB‐SEM)

hSC seeded onto CTEs were fixed in 4%PFA for 30 min, followed by fixation in 2.5% glutaraldehyde in 0.1 M sodium cacodylate buffer overnight at 4°C, with subsequent washes in sodium cacodylate buffer. A quenching solution containing 20 mm glycine (Merck, Millipore) was applied for 20 min on ice, before initiating the ROTO protocol sample preparation. Briefly, samples were post‐fixed in a reduced osmium solution of 1% OsO_4_/1% potassium ferrocyanide for 1 h at 4°C, in the dark, washed in distilled water and then incubated in 1% Thiocarbohydrazide (TCH, EMS) for 30 min at room temperature. After rinsing in water, the specimens were incubated in 1% OsO_4_ aqueous solution for 30 min in the dark at room temperature. This was followed by en‐bloc staining with a 1% uranyl acetate solution, overnight at 4°C. After washing in deionized water, the samples were immersed for 3 min in 0.15% tannic acid solution. Dehydration was then initiated in an ascending series of ethanol (30%; 50%; 70%; 2% × 95%; 3% × 100%, Merck) with each step lasting 30 min on ice. The samples were embedded in a mixture of absolute ethanol/low viscosity resin in the following ratios: 2:1; 1;1; 1;2 and then embedded in absolute resin overnight, followed by three changes of resin, each lasting 2–3 h. The excess resin was removed by washing with absolute ethanol. The specimens were incubated in an oven at 70°C for 24 h to polymerize, mounted on a 12 mm pin aluminum stub (Agar Scientific) with silver paste (RS components) and finally sputtered with a 30 nm layer of gold using the HR208 sputter coater (Cressington). The Focused Ion Beam Scanning Electron Microscope Helios 5CX (FIBSEM, Thermofisher) was used to mill and image each sample. Milling was performed in a region of interest measuring 50 × 15 µm at 30KV with a current of 2.5 nA for regular cross‐sectioning and 0.43 nA for cleaning cross‐sectioning. Cross‐section imaging was conducted using SEM in through‐the‐lens, backscattered mode with a magnification range from 1 to 30 kx.

### 2.7 Manufacturing of Connective Tissue Equivalent (CTE) and hSC Seeding

The workflow for the fabrication and manipulation of the bioengineered hSC‐instructive microenvironment involves two main steps: (i) the fabrication of the CTE and (ii) its subsequent culture with primary hSCs. For the fabrication of CTE, porous gelatin microbeads were used as a temporary scaffold to support the adhesion and proliferation of hDFs, leading to the formation of µTPs within a spinner flask within 9–11 days. This dynamic culture system provided a controlled environment that facilitated cell‐matrix interactions and micro‐tissue precursors (µTPs) development. Porous gelatin microbeads were fabricated using an optimized water‐in‐oil‐in‐water (O/W/O) double emulsion technique and crosslinked with 4% w/w glyceraldehyde (cat. #G5001, Merck). The µTPs were then transferred into a maturation chamber, within silicon molds (1 mm thick, 8 mm in diameter) and further cultured in a spinner flask bioreactor, at 60 rpm for 6 weeks, promoting tissue maturation and compaction into CTE. The culture medium, supplemented with 0.5 mm ascorbic acid (AA, 2‐O‐α‐D‐glucopyranosyl‐L‐ascorbic acid, TCI), was refreshed every 3 days. After opening the chambers, the samples were transferred to six‐well low‐attachment multiwells and incubated overnight in complete medium without AA. On the following day, the seeding process began. The culture media was removed, and the samples were allowed to dry before being placed in a 60 mm Petri dish. A 5 µL drop of fibronectin (cat. # F0895, Merck), prepared at 50 µg/mL by diluting the sterile stock solution 1:20 in PBS, was added to each sample. The fibronectin incubation lasted for a minimum of 40 min at 37°C, followed by an additional 10 min at room temperature (RT). At this stage, any excess of fibronectin was expected to have dried. The samples were then transferred into a 24‐well transwell plate and seeded with a drop of hSC (500 000 cells in 15 µL of cell culture medium) or with eight spheroids (each of them 60 000 cells) and incubated for 3 h. After this incubation period, the samples were submerged in culture medium (DMEM/F12 supplemented with 10% FBS, 1% P/S, 2% NEAA) and left undisturbed for 3 days. On the fourth day, the samples were transitioned to an air‐liquid interface (ALI) culture to promote epithelial polarization, enhance oxygen exchange, and improve cell polarity. From this point onward, the apical side of the transwell was left without medium to maintain the ALI conditions. The medium used for this culture was DMEM/F12 supplemented with 10% fetal bovine serum (FBS), 1% penicillin/streptomycin (P/S), 2% non‐essential aminoacids (NEAA) + 100 ng/mL FSH and 10 µM testosterone. CTE‐hSC and CTE‐hSC‐sph culture was performed up to 28 days.

### 2.8 Immunofluorescence Staining

For immunofluorescence analysis on cells and spheroids, at day 3 of culture samples were fixed in 4% paraformaldehyde (PFA) for 30 min at room temperature RT, followed by washing with 1X phosphate‐buffered saline (PBS). Permeabilization was then performed using 0.1% Triton X‐100 (Merck, Italy) in PBS for 5 min at RT, followed by an additional PBS wash. Samples were blocked with 1% bovine serum albumin (BSA) for 1 h at RT and incubated overnight at 4°C with primary antibody against SOX9 (rabbit monoclonal antibody, cat. #A19710, CliniScience). Subsequently, samples were incubated for 1 h at RT with the Alexa Fluor 488‐conjugated goat anti‐rabbit IgG (H + L) secondary antibody (1:500, Invitrogen, Italy), followed by 1 h incubation with LipidSpot 488 Lipid Droplet Stain (1:1000, cat. # 70065, Cliniscience). Samples were washed with PBS and nuclear staining was performed with 4′,6‐diamidino‐2‐phenylindole (DAPI) or Hoechst (1:10000, Merck, Italy) for 20 min at RT. Samples were imaged using a confocal laser scanning microscope (Leica TCS SP5 II). Z‐stacks were acquired at multiple locations within the samples with a 12‐bit resolution, a 1024 × 1024 pixel format, and either a 10× or 25× water‐immersion objective. Z‐projections were subsequently analyzed using ImageJ software (Fiji). For each biological replicate, at least five independent fields per sample were acquired and analyzed.

### 2.9 RNA Extraction and PCR

Total RNA extraction was performed using the TRIzol‐chloroform method followed by column‐based purification. Samples were placed on ice, and 500 µL of TRIzol reagent (Life Technologies) was added to each Eppendorf tube containing the sample. The samples were disrupted using a customized pestle, obtained with an ASIGA Direct light processing (DLP) printer (MAX X27), and vortexed, followed by 5 min incubation at RT. Subsequently, 100 µL of chloroform (CHCl_3_) was added, vortexed, and incubated for 5 min at RT. The samples were centrifuged at 12 000 g for 15 min at 4°C. After centrifugation, the temperature was set to RT, and the upper aqueous phase (∼300 µL) was carefully transferred to a new Eppendorf tube. An equal volume of 100% ethanol was added and mixed thoroughly. The entire volume was then transferred to a Zymo‐Spin IC column fitted with a collection tube (Aurogene). The columns were centrifuged at 16 000 g for 30 s at RT, and the flow‐through was discarded. Next, 400 µL of RNA wash buffer was added to the column, followed by centrifugation at 16 000 g for 30 s at RT, and the flow‐through was discarded. For DNA removal, an RNA reaction mix was prepared by combining 5 µL of DNase I (Merck) with 35 µL of DNA digestion buffer. 40 µL of this mix was applied directly to the membrane of the column, and the column was incubated for 15 min at RT without centrifugation. Following incubation, 400 µL of RNA Prep buffer was added, and the column was centrifuged at 16 000 g for 30 s at RT, discarding the flow‐through. Subsequently, 700 µL of RNA Wash buffer was added, centrifuged at 16 000 g for 30 s at RT, and the flow‐through was discarded. An additional 400 µL of RNA Wash buffer was added, and the column was centrifuged at 16 000 g for 1 min at RT. The column was then transferred to a new Eppendorf tube, and 12 µL of DNase/RNase‐free water was added to elute the RNA. Finally, the column was centrifuged at 16 000 g for 30 s at RT, and the RNA‐containing eluate was collected. RNA concentration and purity was measured with a Multiskan SkyHigh Microplate Spectrophotometer.

cDNA synthesis from the extracted total RNA was performed using the iScript CDNA synthesis kit (BioRad) following the manufacturer's protocol. Each reaction was carried out with 500 ng of RNA per sample. Reverse transcription was conducted using a 2720 Thermal Cycler (Applied Biosystems, US) with the following cycling conditions: 5 min at 25°C, 20 min at 46°C, 1 min at 95°C. The resulting cDNA samples were then stored at 4°C until further use. Polymerase chain reaction (PCR) was performed using SYBR Green qPCR Mastermix (BioRad) along with pre‐designed primers listed in Table 1. Real‐time PCR was performed using BIORAD CFX96 Real‐Time system C100 Touch Thermal cycler (Biorad, US) following a 40‐cycle, three‐step protocol: denaturation at 95°C for 10 s, annealing at 60°C for 30 s, and elongation at 72°C for 15 s. Gene expression was normalized to the GAPDH housekeeping gene and analyzed using the 2^−ΔΔCt^ method, with day 0 control samples serving as calibrators. Day 0 corresponds to the starting hSC population collected at the time of seeding, prior to culture within the CTE system and before exposure to long‐term ALI conditions. Each reaction well contained 1.5 µL of cDNA.

**TABLE 1:** List of target genes and corresponding forward and reverse primers used for quantitative real‐time PCR (qRT‐PCR) analysis. The table includes primers for key Sertoli cell markers, including GATA4, CLDN11, ABP, SOX9, FSHR, ZO‐1, and reference genes such as GAPDH.

Target mRNA	Forward primer	Reverse primer
GATA4	GAAGGAGCCAGCCTAGCAG	GAAAACGACGGCAACAACGA
CLDN11	CTGGCTGGTGTTTTGCTCAT	CATGAGTCGGGGAGCTAGAG
ABP	CCATCTTGGCTCAGTCTCCA	GGGGTTCTTAGGTGGAGCTT
SOX9	ATGAAGATGACCGACGAGCA	AACTTGTCCTCCTCGCTCTC
FSHR	GGCCATGCTCATCTTCACTG	ATAGAGGAAGGGGTTGGCAC
ZO‐1	GCGGTCAGAGCCTTCTGATC	CATGCTTTACAGGAGTTGAGACAG
GAPDH	CCACCCATGGCAAATTCCATGGCA	TCTAGACTGGCAGGTCAGGTCCACC

### 2.10 Sample Preparation for Immunohistochemistry

At DIV3, DIV7, DIV14, DIV28 samples were fixed in formalin (10% for 30 min to 1 h at RT) and dehydrated through a series of ethanol solutions with increasing concentrations. The dehydration was performed sequentially in 75% ethanol for 30 min, followed by 85% ethanol for 30 min, then 95% ethanol for 30 min, and finally in two consecutive steps of 100% ethanol, each for 30 min. Samples were stored in at 4°C or further processed for paraffin embedding. For paraffin embedding, routine histological procedures were performed and paraffined blocks were sectioned in 7 µm‐thick slices with a microtome.

### 2.11 Histological Staining on Tissue Slices

Prior to staining, the tissue sections underwent deparaffinization, which involved immersing them in two successive xylene baths for 20 min each. This was followed by a series of ethanol treatments with gradually decreasing concentrations (100%, 95%, 85%, and 75%, each for 10 min), concluding with a 10‐min rinse in water. For hematoxylin and eosin (H&E) staining (Bio Optica), the samples were first incubated in hematoxylin for 15 min to stain the nuclei, then rinsed under running water. Next, eosin staining was applied for 5 min to target the cytoplasm and extracellular matrix. Finally, the slides were mounted with DPX (Merck) and left to dry overnight in a fume hood. For Masson's Trichrome staining (Bio Optica), three dyes were used: hematoxylin, fuchsin, and aniline. The process began with 15 min of hematoxylin staining, followed by a rinse in distilled water (dH_2_O). The samples were then treated with fuchsin for 5 min, followed by another water rinse. Subsequently, they were incubated for 7 min in a mordant solution containing phosphomolybdic and phosphotungstic acids (1:1) diluted in water. Without additional washing, the samples were then stained with aniline for 10 min. To complete the process, the slides underwent dehydration by immersion in an ethanol gradient (75%, 85%, 95%, and 100%), followed by xylene baths. Finally, the slides were mounted with DPX (Merck) and left to dry overnight in a fume hood. Images were acquired with Olympus BX53 light microscope with 10x, 20x, and 40x objective lenses.

### 2.12 Immunofluorescence on Tissue Slices

The samples underwent deparaffinization through two successive xylene washes of 3 and 10 min each, followed by rehydration in a graded ethanol series (100%, 100%, 95%, 95%, 70%, 50%, 30%) and a final rinse dH_2_O. From this point onward, the slides were kept in a moist environment. For immunofluorescence staining, the slides were washed twice in PBS (2 min each) before permeabilization with 0.2% Triton X‐100 for 5 min, followed by an additional two PBS washes (2 min each). To enhance antigen exposure, the tissue sections were placed in 0.001 M citrate buffer (pH 6), diluted 1:10 in distilled water, and sealed in a histology tray with cling film. The slides were then subjected to microwave heating: 4 min on defrost mode, followed by two low‐power 5‐min cycles, with short intervals to prevent overheating. After heating, the slides were cooled in the buffer at room temperature (RT) for 45 min, washed in PBS, and outlined using a PAP‐PEN barrier. Blocking was performed by incubating the slides in histoblock solution (6% BSA, 5% FBS, 20 mm MgCl_2_, 0.2% Tween20 in PBS) for 2 h at RT in a humid chamber. The blocking solution was then removed, and the slides were incubated overnight at 4°C with primary antibodies SOX9, ZO‐1, and Occludin (OCLDN) (Rabbit monoclonal antibodies, Cliniscience) (1:200 in histoblock solution) or α‐SMA (mouse polyclonal antibody, Abcam), followed by two PBS washes. Next, the slides were incubated with Alexa Fluor 488‐conjugated goat anti‐rabbit IgG (H + L) secondary antibody (1:500 in histoblock solution, Invitrogen, Italy) or Alexa Fluor 555‐conjugated goat anti‐mouse IgG (H+L) secondary antibodies (1:500, Invitrogen, Italy) for 1 h at RT in a humid chamber, followed by a 5‐min PBS wash. Nuclear staining was performed using DAPI (1:1000 in PBS) for 20 min at RT in a humid chamber, followed by another 5‐min PBS wash. Finally, the slides were mounted using 80% glycerol in PBS and sealed with clear nail polish after a brief drying period. Images were obtained as explained in section 2.4. The acquired images underwent semi‐quantitative analysis in ImageJ (Fiji), where the signal fraction per cell was measured. To achieve this, SOX9‐, ZO‐1‐, α‐SMA‐, and OCLDN‐positive areas were identified by thresholding and quantified. The resulting positive area was normalized to the total cell number, determined from the DAPI channel using the “Analyze Particles” function in ImageJ. For each biological replicate, at least three sections per sample and 5–8 randomly selected fields per section were analyzed for each condition. The quantification therefore reflects total signal area per cell. Data were pooled per biological replicate prior to statistical analysis. Membrane‐associated distribution of junctional markers was evaluated qualitatively by image inspection and used to support the interpretation of junctional organization. Statistical analysis was performed as described in section 2.13.

### 2.13 Statistical Analysis

All quantitative data are presented as mean ± standard deviation (SD), unless otherwise stated. Biological replicates correspond to two independent lots of human Sertoli cells (hSCs) purchased from ScienCell. For each biological replicate, technical replicates (*n* = 3) were performed depending on the specific assay. For gene expression analysis, qPCR reactions were performed in technical triplicate for each biological replicate. For immunofluorescence quantification, multiple fields and sections per condition were analyzed for each biological replicate, as detailed in the corresponding Methods sections. Statistical analyses were performed using GraphPad Prism (GraphPad Software, USA). Comparisons between two groups were performed using an unpaired two‐tailed Student's *t*‐test, while multiple group comparisons were analyzed using one‐way ANOVA followed by appropriate post hoc tests, as specified in the figure legends. A *p*‐value < 0.05 was considered statistically significant.

## 3 Results

### 3.1 Optimization of hSC Culture Conditions

**FIGURE 1:**
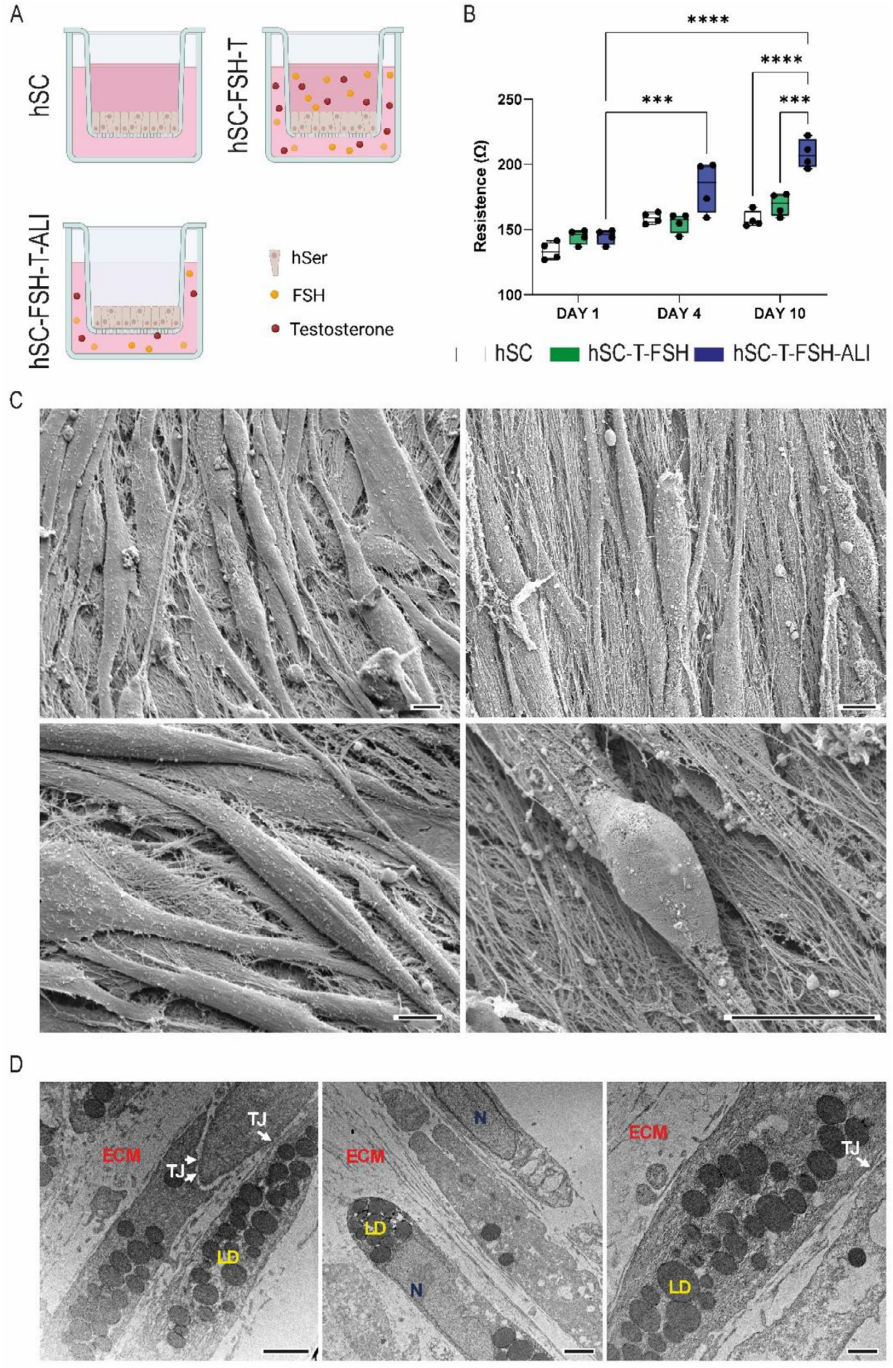
Optimization of hSC Culture Conditions in 2D Transwell System. (A) Schematic representation of the three culture conditions used for primary hSCs: (i) hSC: basal medium only; (ii) hSC‐FSH‐T: stimulation with FSH and T. (iii) hSC‐FSH‐T‐ALI: cells exposed to FSH and T under an air‐liquid interface (ALI) condition. (B) Trans epithelial electrical resistance (TEER) measurements over time, comparing different culture conditions: hSC (control), hSC‐T‐FSH (treated with testosterone and FSH), and hSC‐T‐FSH‐ALI (treated with testosterone and FSH under ALI conditions). Statistics: ****p < 0.0001, *p < 0.001. (C) SEM images of hSC‐T‐FSH‐ALI cultured in 2D Transwell inserts, showing cellular morphology and interaction with the substrate. Scale bars: 10 µm. (D) TEM images of hSCs‐T‐FSH‐ALI, highlighting extracellular matrix (ECM, red), tight junctions (TJ, white arrows), nuclei (N, blue), and lipid droplets (LD, yellow). Scale bars: 2 µm (left image), 1 µm (central and right image).

To optimize culture conditions for hSCs in a 2D Transwell system, we evaluated their morphology, barrier properties, and ultrastructural features under different conditions (Figure 1). Barrier integrity was assessed via TEER measurements at different time points (DIV 1,4,7). A significant increase in TEER values was observed in hSCs cultured with FSH and Testosterone (hSC‐FSH‐T), with an even greater enhancement under ALI conditions (hSC‐FSH‐T‐ALI) (*p* < 0.0001), indicating improved barrier function in response to hormonal stimulation and ALI exposure. These results indicate that hormonal stimulation and ALI exposure enhance the barrier function of hSC monolayers (Figure 1. SEM images revealed that hSCs adhered to the fibronectin‐coated Transwell membrane, exhibiting an elongated morphology with extensive cytoplasmic extensions.TEM further confirmed these findings, showing the presence of well‐formed tight junctions (white arrows), which are crucial for maintaining the blood‐testis barrier. Additionally, lipid droplets (yellow) were observed in the cytoplasm, suggesting active metabolic activity and lipid storage, which are characteristic features of mature Sertoli cells. The presence of extracellular matrix (ECM, red) and distinct irregularly shaped nuclei (N, blue) further indicated a well‐organized cellular architecture.

### 3.2 Characterization of hSC and hSC‐sph

**FIGURE 2:**
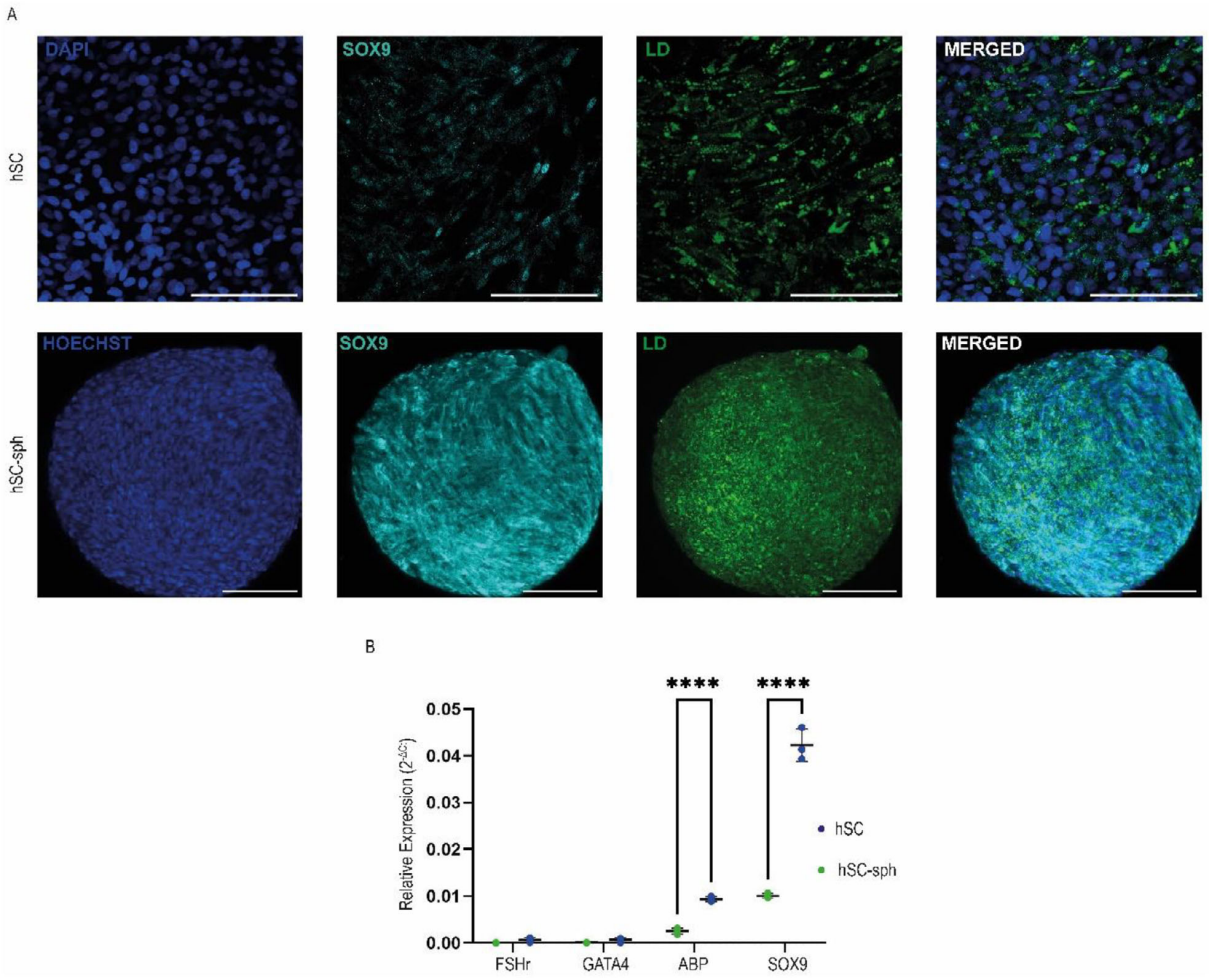
Characterization of hSC and hSC‐sph within a CTE system. (A) Immunofluorescence analysis of hSCs and hSC‐sph at day 3. Fluorescence images show monolayer hSCs (top) and hSC‐sph (bottom) stained for nuclei (DAPI/Hoechst, blue), SOX9 (green, Sertoli cell marker), and Live/Dead (LD, green, viability assay). Merged images display channel overlays. Scale bars: 100 µm (hSC); 200 µm (hSC‐sph). (B) Gene expression analysis. RT‐PCR quantification of FSHr, GATA4, ABP, and SOX9 expression in hSCs and hSC‐sph. Statistics: ****p < 0.0001, *n* = 3.

To assess the morphological, metabolic, and functional differences between monolayer hSCs and hSC‐sph at day 3, immunofluorescence staining and gene expression analysis were performed. Nuclei were labeled using DAPI (hSC) or Hoechst (hSC‐sph) (blue), while SOX9 (cyan), a key SC transcription factor, was used to confirm Sertoli cell identity. Lipid droplets (LDs), a metabolic hallmark of SC, were visualized in green as an indicator of lipid storage and cellular activity, both of which are essential for Sertoli cell function (Figure 2A) [27]. In 2D monolayer culture, hSCs exhibited a spread‐out, elongated morphology, with SOX9 expression distributed throughout the cytoplasm, suggesting a less organized phenotype. In contrast, when cultured under ultra‐low attachment conditions, hSCs spontaneously aggregated into spheroids, forming compact 3D structures. Within these aggregates, SOX9 staining was more intense and homogenously distributed, suggesting enhanced maintenance of the Sertoli cell phenotype in 3D culture. Furthermore, LDs accumulation appeared qualitatively higher in hSC‐sph compared to 2D cultures, indicating a tendency toward increased lipid storage in the spheroid condition [27]. Merged images highlighted these differences, emphasizing the enhanced cellular organization, metabolic activity, and SC identity in spheroid culture. To further investigate the functional differences between hSC and hSC‐sph, quantitative RT‐PCR was performed to analyze the expression levels of FSHr, GATA4, ABP, and SOX9 (Figure 2B). While FSHr and GATA4 expression remained low and comparable between the two conditions, a significant upregulation of ABP (androgen‐binding protein) and SOX9 was observed in hSC‐sph compared to monolayer hSCs (****p < 0.0001). The increased expression of ABP suggests enhanced androgen responsiveness, which is essential for Sertoli cell‐mediated support of spermatogenesis [28]. Additionally, the upregulation of SOX9, a crucial transcription factor for Sertoli cell differentiation and function, further supports the hypothesis that 3D spheroid culture promotes a more physiologically relevant Sertoli cell phenotype. The increased accumulation of lipid droplets and upregulation of Sertoli cell markers in spheroid culture suggest that hSC‐sph provide a more physiologically relevant microenvironment compared to monolayer hSC cultures, improving metabolic activity and functional gene expression.

### 3.3 Characterization of hSC in CTE Constructs

**FIGURE 3:**
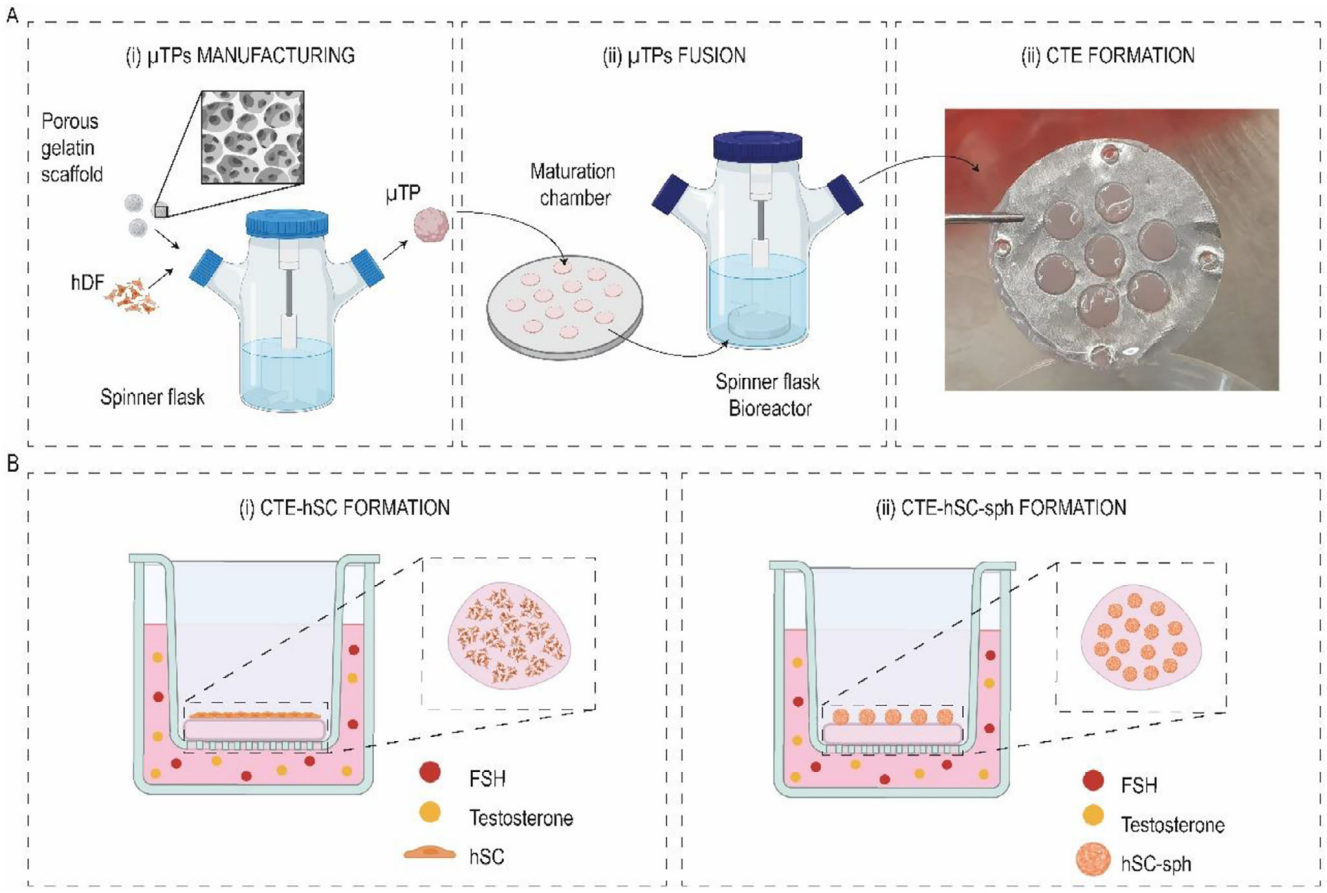
hSC and hSC‐sph within a CTE system. (A) CTE fabrication using a bottom‐up approach, which involves: (i) manufacturing of µTP within a spinner flask, by seeding hDF onto porous gelatin microbeads; (ii) fusion of µTP within a maturation chamber in silicon molds; (iii) obtainment of CTE. (B) Seeding of CTE with: (i) hSC (CTE‐hSC) or with (ii) hSC‐sph (CTE‐hSC‐sph). Samples were cultured within a transwell in optimized culture medium (supplemented with FSH and Testosterone).

To investigate hSC differentiation and structural organization in CTE constructs (Figure 3), we analyzed samples at DIV7 and DIV14.

**FIGURE 4:**
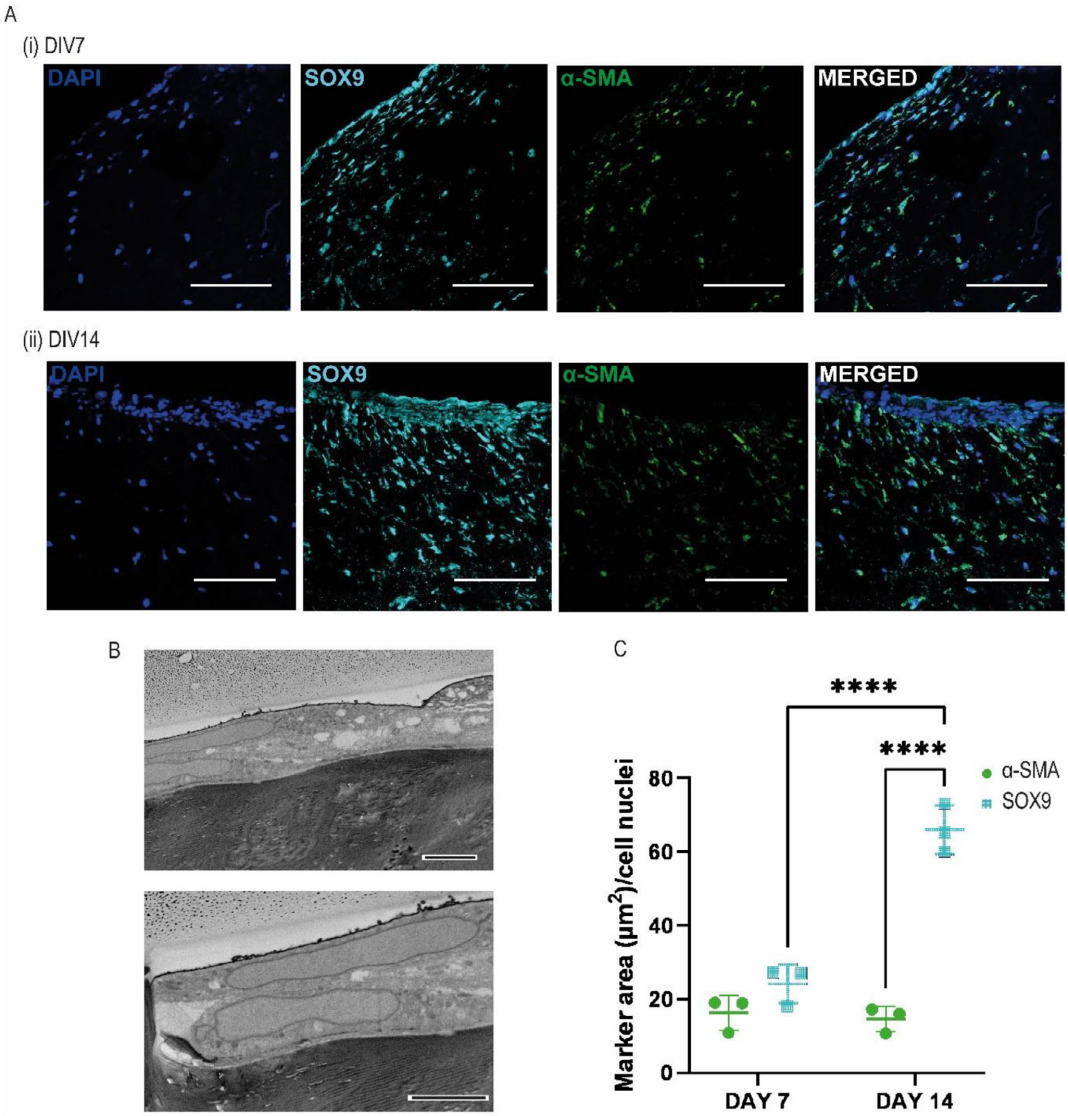
Characterization of hSC in CTE at DIV7 and DIV14. (A) Immunofluorescence staining of SOX9 and α‐SMA in CTE‐seeded cells at DIV7 (i) and DIV14 (ii). DAPI (blue) stains cell nuclei, SOX9 (cyan) marks human Sertoli cells (hSC), and α‐SMA (green) identifies myofibroblasts. Merged images show the spatial distribution of both cell types within the connective tissue equivalent (CTE). Scale bars: 100 µm. (B) FIB‐SEM images at DIV14, showing stratified cellular layers above the CTE structure. Scale bars: 5 µm. (C) Quantification of SOX9+ and α‐SMA+ cells at DIV7 and DIV14. Statistics: ****p < 0.0001.

**FIGURE 5:**
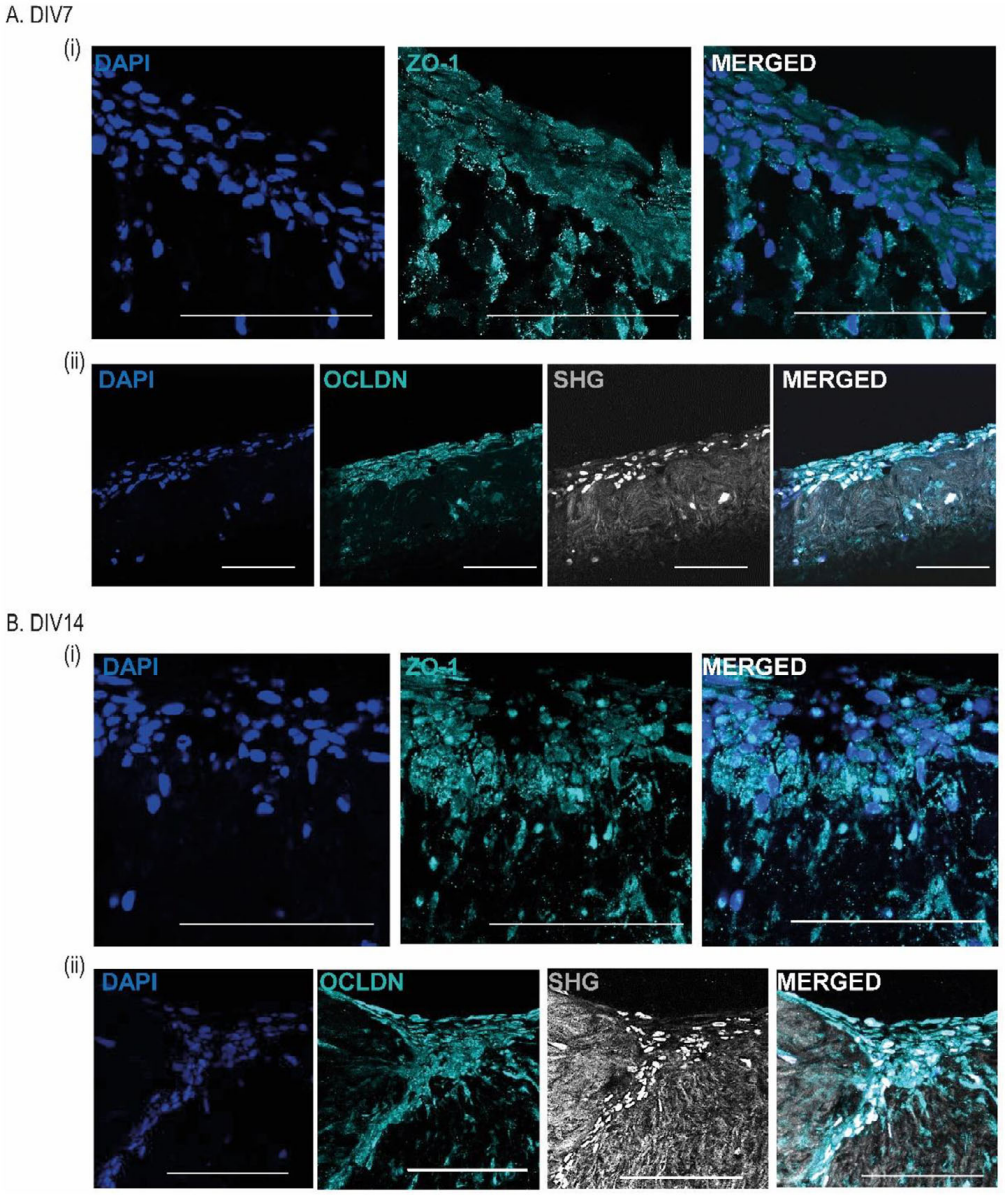
Immunofluorescence Analysis of CTE‐seeded Cells at DIV7 and DIV14. (A) Immunofluorescence staining at DIV7. (i) ZO‐1 (cyan), a tight junction marker, is localized at cell–cell junctions, with DAPI (blue) marking nuclei. (ii) OCLDN (cyan) and SHG imaging visualize tight junctions and fibrillar collagen structures, respectively. Merged images highlight their spatial organization. (B) Immunofluorescence staining at DIV14. (i) ZO‐1 (cyan) localization in cells stratified above the CTE, with DAPI (blue) staining nuclei. (ii) OCLDN (cyan) and SHG imaging, showing enhanced junction formation and collagen organization at later time points. Scale bars: 100 µm.

Immunofluorescence staining revealed a significant increase in SOX9+ cells at DIV14 compared to DIV7 (*p* < 0.0001), indicating that the culture conditions supported hSC expansion while maintaining their identity. In contrast, the α‐SMA^+^ cell population remained stable over time, suggesting that hDFs did not undergo significant proliferation. This phenotype is consistent with peritubular myoid‐like cells, which naturally express α‐SMA and contribute to the testicular stromal compartment (Figure 4A,C). FIB‐SEM imaging further confirmed progressive cellular integration within the CTE, showing the formation of stratified cellular layers at DIV14 (Figure 4B). These results demonstrate that hormonal stimulation and ALI culture conditions promote Sertoli cell expansion while maintaining their phenotypic identity. To assess junctional maturation and tissue remodeling, ZO‐1 and OCLDN immunostaining were performed at both time points. At DIV7, ZO‐1 and OCLDN appeared diffusely distributed, consistent with an early, immature phase of BTB assembly. SHG imaging at this stage revealed minimal ECM reorganization and sparse fibrillar collagen. By DIV14, however, a more organized ZO‐1 network was observed at hSC‐hSC interfaces, accompanied by increased OCLDN expression and a denser collagen architecture, indicating progressive tight junction maturation and ECM remodeling (Figure 5). Given the more advanced structural and functional features observed at DIV14, this time point was selected as the starting condition for subsequent spheroid‐based model analyses.

### 3.4 Histological Characterization of CTE‐hSC and CTE‐hSC‐sph Over Time

To assess the structural and ECM composition of CTE seeded with either human SC (CTE‐hSC) or human Sertoli cell spheroids (CTE‐hSC‐sph) over time (as reported in Figure 3), histological analyses were performed at days 14, 21, and 28 using Hematoxylin & Eosin (H&E) and Masson's Trichrome (MT) staining. For CTE‐hSC, at DIV14, H&E staining revealed a dispersed distribution of hSCs within the tissue, with cells loosely integrated into the construct. MT staining showed early ECM deposition, with collagen fibers beginning to form around the cells (Figure 6A). At DIV21, H&E staining indicated increased cellular organization and tissue compaction, while MT staining highlighted a denser collagen matrix, suggesting ongoing ECM remodeling (Figure 6B). By DIV28, H&E images showed further cell aggregation and the presence of vacuole‐like structures, while MT staining confirmed enhanced collagen deposition and a more structured ECM (Figure 6C). For CTE‐hSC‐sph, histological analysis at DIV14 showed that hSC‐sph remained largely intact within the tissue, maintaining their spherical structure, as observed in H&E‐stained sections (Figure 6D). MT staining demonstrated collagen deposition surrounding the spheroids, suggesting initial matrix integration. At DIV21, H&E images showed greater interaction between spheroids and the surrounding tissue, with evidence of partial spheroid fusion. MT staining revealed more pronounced collagen deposition, indicative of ECM remodeling (Figure 6E). By DIV28, H&E staining highlighted a well‐integrated tissue with embedded spheroids, while MT staining confirmed a highly organized collagen network, suggesting advanced maturation and structural stability (Figure 6F). These findings demonstrate that both CTE‐hSC and CTE‐hSC‐sph undergo progressive tissue remodeling and ECM deposition over time. In the spheroid‐seeded condition, cells maintained greater structural cohesion within the construct and showed a more compact and integrated spatial arrangement, together with closer association with the surrounding remodeled matrix. These features suggest that 3D aggregation may favor cell retention and structural organization within the construct.

FIGURE 6Histological analysis of CTE‐hSC and CTE‐hSC‐sph over time. Hematoxylin & Eosin (H&E) and Masson's Trichrome (MT) staining of CTE seeded with human Sertoli cells (CTE‐hSC) or human Sertoli cell spheroids (CTE‐hSC‐sph) at DIV14 (A,D), DIV21 (B,E), and DIV28 (C,F). (i) H&E staining shows cell distribution and tissue organization, while (ii) MT staining highlights ECM remodeling and collagen deposition (blue).

**Figure:**
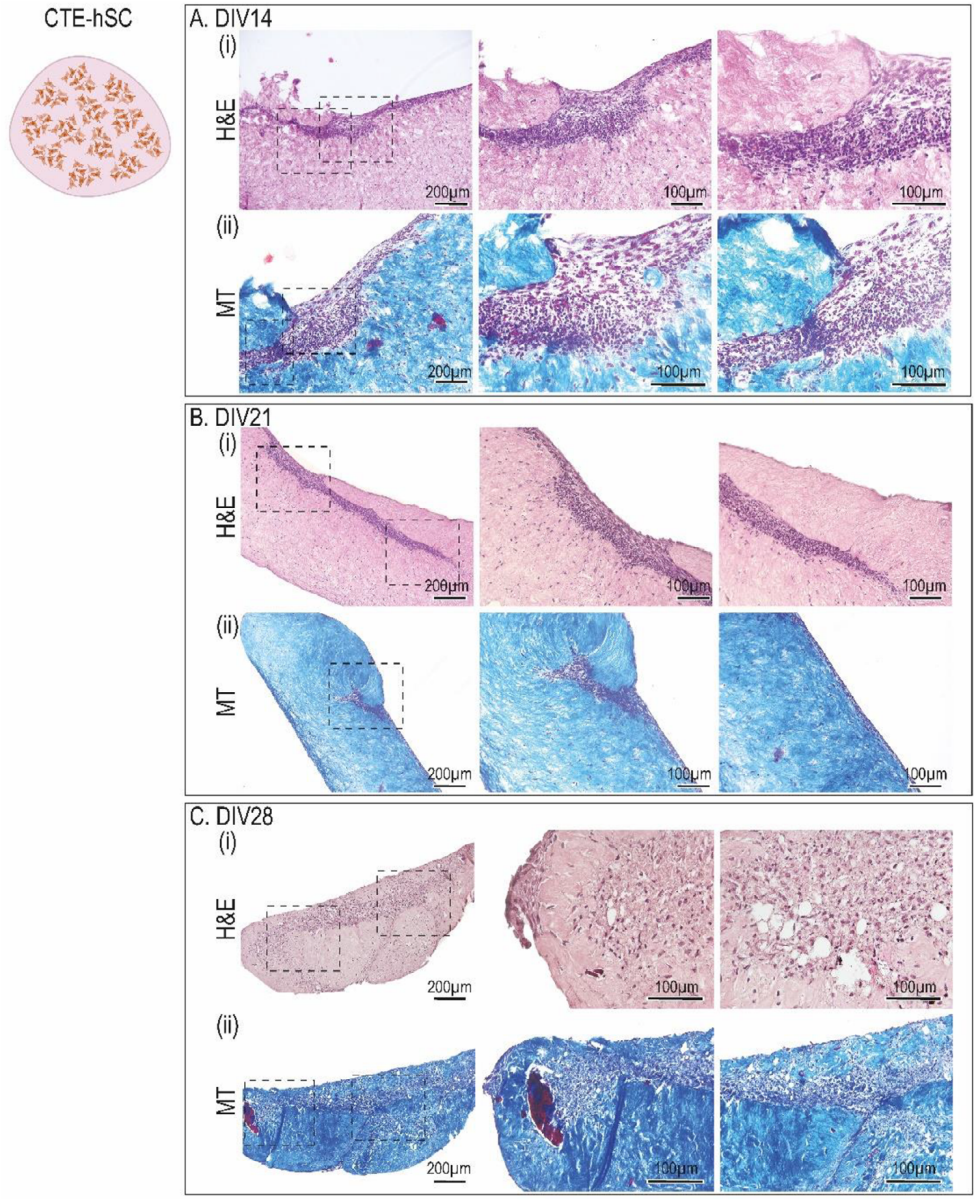

**Figure:**
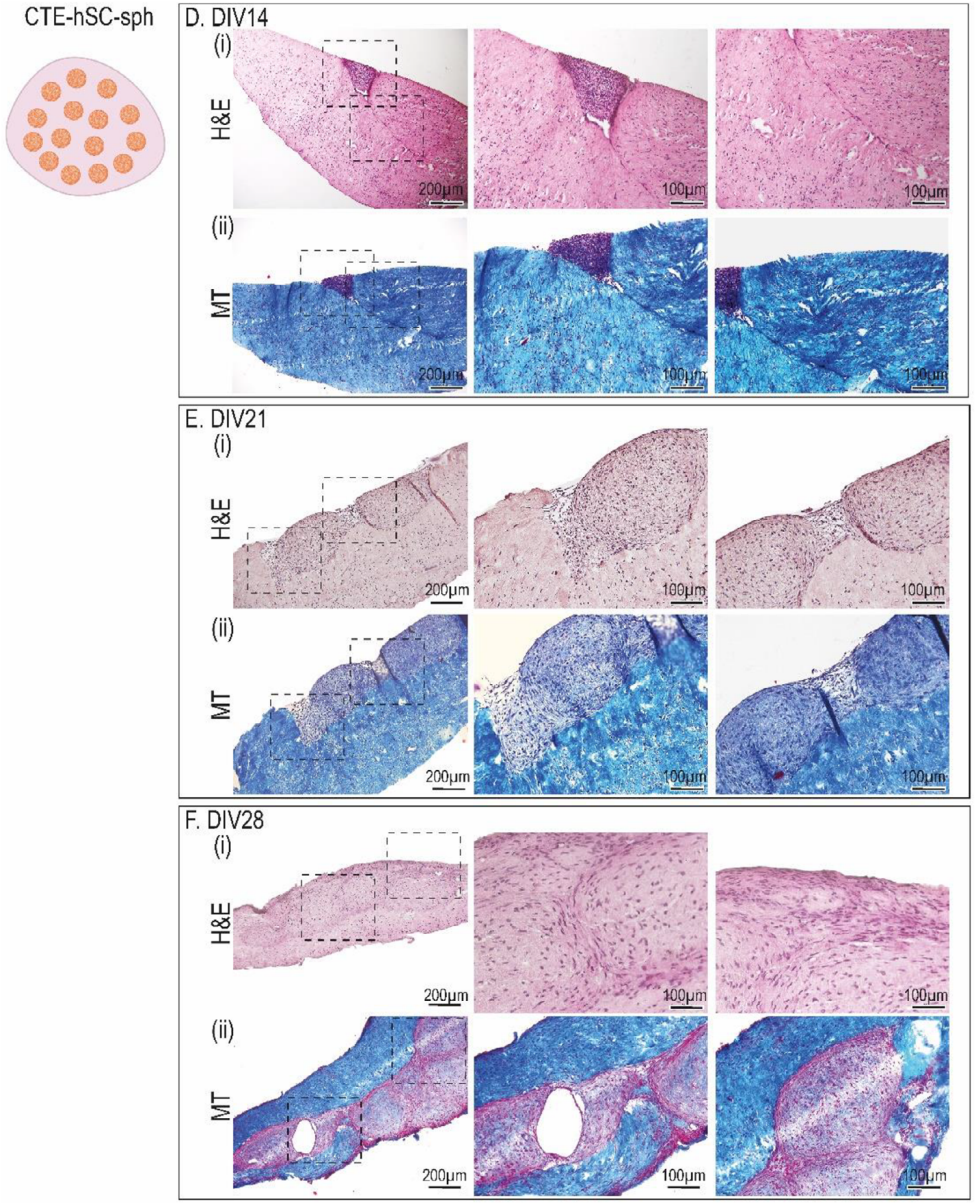

### 3.5 Immunofluorescence and Gene Expression Analysis of CTE‐hSC and CTE‐hSC‐sph

**FIGURE 7:**
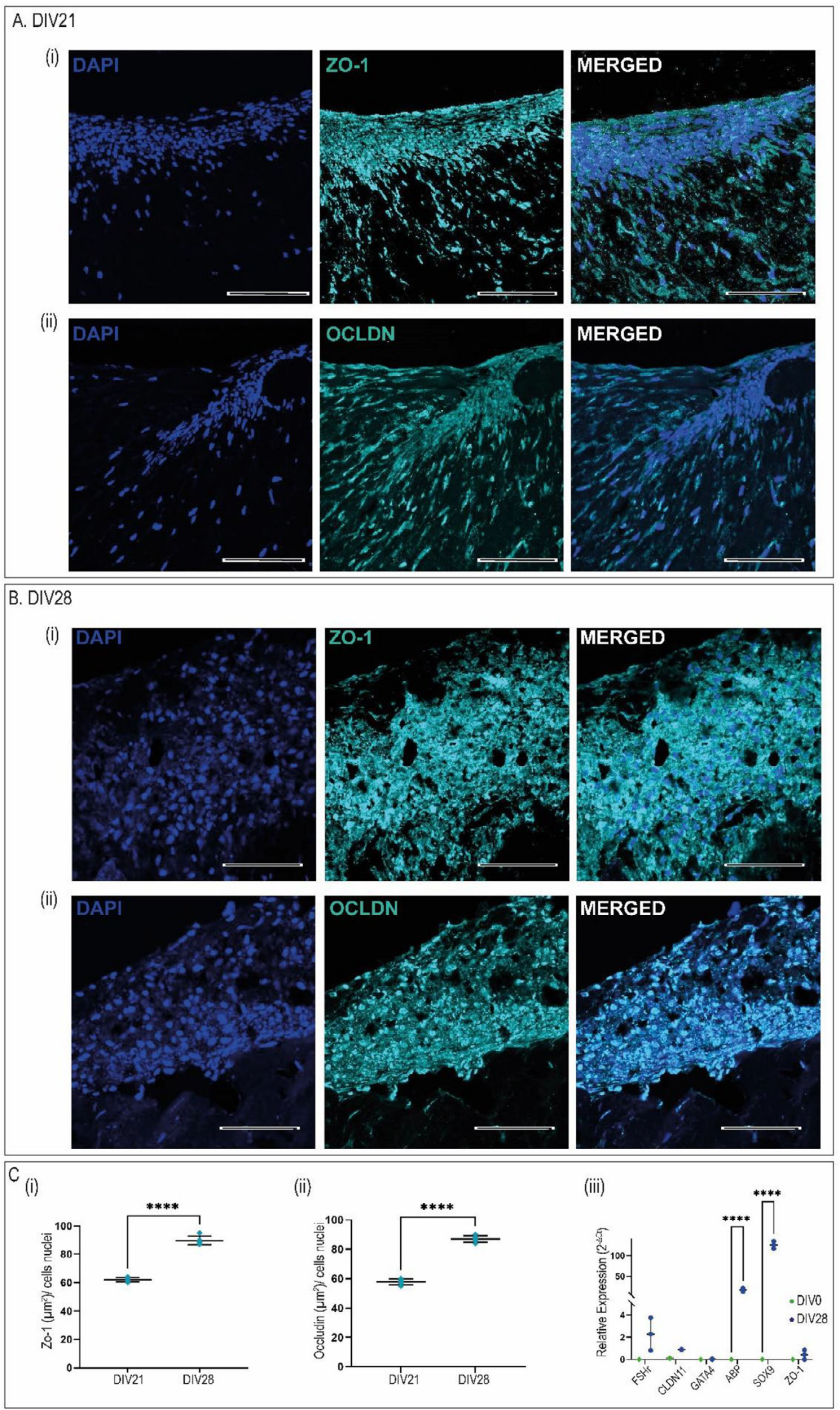
Immunofluorescence and gene expression analysis of CTE‐hSC. (A, B) Immunofluorescence staining of tight junction markers ZO‐1 (i) and OCLDN (ii) at DIV21 (A) and DIV28 (B). DAPI (blue) stains nuclei, while ZO‐1 and OCLDN (cyan) highlight tight junction localization. Merged images show protein distribution. Scale bars: 200 µm. (C) Quantification and gene expression analysis: (i, ii) quantification of ZO‐1 and OCLDN expression (µm^2^ per cell nucleus) at DIV21 and DIV28; (iii) RT‐qPCR gene expression analysis of FSHr, CLDN11, GATA4, ABP, SOX9, and ZO‐1 at DIV0 and DIV28. Statistics: **p < 0.0001, *n* = 3.

Since significant morphological differences between CTE‐hSC and CTE‐hSC‐sph emerged at 21 and 28 DIV, as observed through histological characterization, we evaluated the tight junctions and the expression of key Sertoli cell markers at these time points. Immunofluorescence staining of ZO‐1 (zonula occludens‐1) and OCLDN (occludin), two essential tight junction proteins, revealed a progressive increase in their localization within the tissue. In CTE‐hSC, at DIV21, ZO‐1 and OCLDN expression appeared more dispersed, with ZO‐1 localized along cell–cell junctions and OCLDN distributed throughout the construct (Figure 7A). By DIV28, both proteins exhibited a more extensive and defined expression pattern, indicating tight junction maturation and enhanced Sertoli cell barrier formation (Figure 7B). Merged images highlight the increasing organization of these proteins within the tissue, suggesting progressive structural integration over time. Quantification of protein expression confirmed this trend, with ZO‐1 and OCLDN levels significantly increasing from DIV21 to DIV28 (****p < 0.0001). Since significant morphological differences between CTE‐hSC and CTE‐hSC‐sph emerged at DIV21 and DIV28, as observed through histological characterization, we evaluated tight junction‐associated proteins and SC‐related markers at these time points. For gene expression analyses, day 0 was defined as the baseline hSC condition at the time of seeding, prior to culture within the CTE construct and before exposure to prolonged ALI conditions. Therefore, fold changes reflect transcriptional variations acquired during culture relative to the initial state of the cells. SOX9, ABP, and ZO‐1 were significantly upregulated at DIV28, while FSHr, CLDN11, and GATA4 remained at low expression levels (Figure 7C).

**FIGURE 8:**
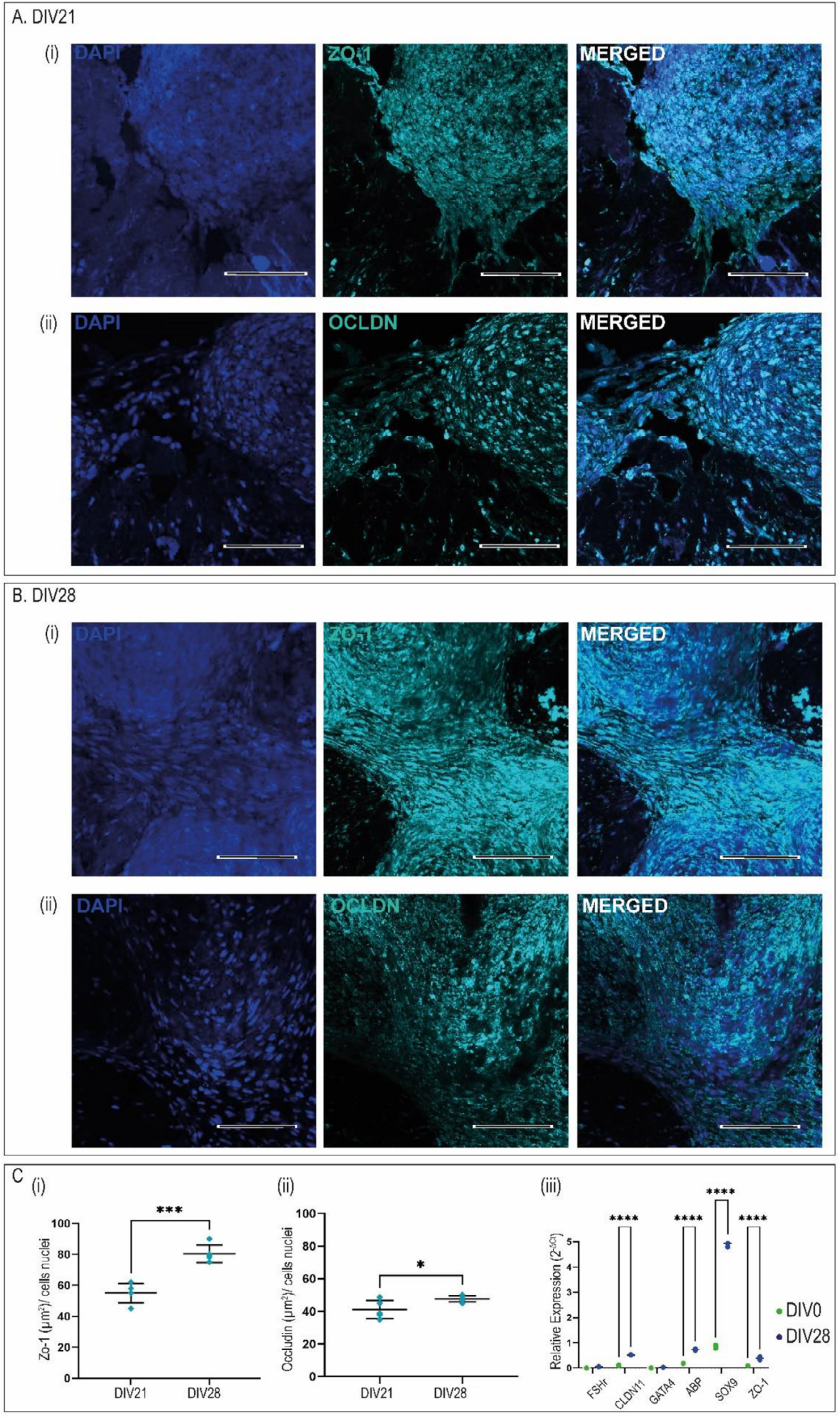
Immunofluorescence and gene expression analysis of CTE‐hSC‐sph. Immunofluorescence staining of tight junction markers ZO‐1 (i) and OCLDN (ii) at DIV21 (A) and DIV28 (B). DAPI (blue) stains nuclei, while ZO‐1 and OCLDN (cyan) highlight tight junction localization. Merged images show protein distribution. Scale bars: 200 µm. (C) Quantification and gene expression analysis: (i, ii) quantification of ZO‐1 and OCLDN expression (µm^2^ per cell nucleus) at DIV21 and DIV28; (iii) RT‐qPCR gene expression analysis of FSHr, CLDN11, GATA4, ABP, SOX9, and ZO‐1 at DIV0 and DIV28. Statistical significance: ****p < 0.0001; ***p < 0.001; *p < 0.5, N = 3.

Similarly, in CTE‐hSC‐sph, immunofluorescence staining of ZO‐1 and OCLDN at DIV21 and DIV28 showed a progressive increase in tight junction protein localization. At DIV21, ZO‐1 and OCLDN were already expressed, though their distribution was less organized (Figure 8A). By DIV28, both markers exhibited more structured localization, suggesting enhanced tight junction formation over time (Figure 8B). Quantification analysis confirmed a significant increase in ZO‐1 (*p < 0.001) and a moderate increase in OCLDN (*p* < 0.05) between DIV21 and DIV28. Gene expression analysis showed significant up‐regulation of FSHr, CLDN11, GATA4, ABP, SOX9, and ZO‐1 at DIV28 (**p < 0.0001) compared to DIV0, indicating an increase in Sertoli cell‐specific markers and tight junction‐related gene expression over time. These findings demonstrate that both CTE‐hSC and CTE‐hSC‐sph exhibit progressive tight junction formation and SC phenotype maintenance with ZO‐1 and OCLDN expression increasing over time in both models. However, gene expression analysis indicates a broader upregulation of SC markers in CTE‐hSC‐sph, suggesting that the spheroid‐based model provides a more favorable context for hSC organization and junction‐associated protein patterning compared to monolayer‐seeded constructs (Figure 8C).

## 4 Discussion

The successful development of a physiologically relevant in vitro model of the testicular niche necessitates not only the presence of SCs, but also the establishment of an appropriate 3D microenvironment capable of sustaining their functionality, guiding ECM remodeling, supporting cell–cell and cell‐matrix interactions, and promoting their self‐organization into tubule‐like structures that recapitulate the architecture of native seminiferous tubules. Although the pivotal role of SC in testicular tissue engineering is well established, faithfully recreating the morpho‐chemical and physical features of the native microenvironment that regulates SC functionality and maturation remains a major challenge [29].

In this work, we addressed this limitation by designing the first ECM‐rich, structurally organized in vitro microenvironment that sustains the functionality and maturation of primary hSCs. We engineered native‐equivalent connective tissue construct seeded with hSC, either as single cells or as spheroids, and investigated their phenotypic evolution, structural organization, tight junction formation, and ECM remodeling over time. Our findings demonstrate that both the biochemical composition and structural features of the microenvironment play a critical role in regulating hSC long‐term maintenance and maturation, which is rarely achieved in vitro.

To establish baseline culture conditions, we first developed a 2.5D Transwell model, which revealed that hSCs responded to hormonal cues (testosterone, FSH) with increased Transepithelial Electrical Resistance (TEER) values and formation of ultrastructural tight junctions, confirming their ability to acquire functional features when exposed to appropriate stimuli (Figure S1) [30]. This preliminary step was essential for defining the optimal culture environment required for hSC function before transitioning to 3D CTE models, where additional spatial constraints and ECM interactions contribute to tissue morphogenesis.

In our model, the transfer of SCs into the CTE initiates a gradual process of cellular infiltration and tissue reorganization, where dynamic ECM remodeling and the still diffuse distribution of junctional proteins, together with the enrichment of SOX9^+^ cells and the reduced fibroblast contribution, characterize an early and still immature phase of niche reconstruction and onset of BTB assembly.

By DIV14, both histological and molecular markers revealed a more mature microenvironment. Masson's Trichrome staining demonstrated increased collagen deposition and matrix compaction, and immunofluorescence analysis revealed that hSCs had not only infiltrated the ECM but also initiated its remodeling, aligning with a progressive upregulation of key SC maturation markers, including SOX9 and ABP. Both CTE‐hSC and CTE‐hSC‐sph exhibited progressive tight junction formation, as reflected by the increasing expression and organization of ZO‐1 and OCLDN over time at the hSC‐hSC interfaces [31]. The upregulation of SC‐specific markers (SOX9, ABP) and BTB proteins (ZO‐1, OCLDN) over time in both configurations confirmed the acquisition of a more differentiated phenotype. It is important to note, however, that SOX9 serves primarily as a maintenance rather than a maturation marker, in line with the use of pubertal cells. The superior performance of the CTE‐hSC‐sph model highlights the importance of initial spatial confinement and cell aggregation in maintaining hSC phenotype. Spheroids preserved structural cohesion and promoted cell–cell communication together with a more organized distribution of junctional proteins. This is consistent with previous organoids studies suggesting that 3D aggregation enhances SC cell–cell signaling and mimics native spatial cues that support BTB assembly [5, 14, 29, 31]. However, although progressive organization of junctional proteins was observed over time, the overall spatial arrangement of hSCs within the construct does not recapitulate the architecture of native seminiferous tubules. Given the absence of additional testicular cell populations, such as peritubular myoid cells and germ cells, as well as of a defined basement membrane organization, a physiologically accurate tissue topology is not expected in this model. Within these constraints, the pre‐existing spheroid architecture may contribute to partial structural organization within the construct and support the progressive organization of junctional proteins [32]. Collectively, our CTE supports gradual and coordinated maturation of human SCs, as reflected by spatial reorganization, TJ formation, and acquisition of functional markers over time. This is particularly relevant when considering that many existing 3D models rely on matrices that can support partial SC organization and junctional assembly, but they often fail to maintain stable phenotypes over time, structural organization, or are unsuitable for translational applications due to poor mechanical integrity or undefined composition [5, 14, 15, 33]. While successful reassembly has been reported in porcine decellularized testicular ECM systems, dECMs derived from human testes present additional challenges. Although they retain native biochemical constituents and can support short‐term cell survival, human testicular cells often fail to achieve full structural and functional reconstitution in vitro. In these systems, hSC commonly cluster on scaffold surfaces without forming polarized seminiferous cord‐like structures, and the BTB fails to fully assemble. Thus, despite enabling short‐term survival and endocrine activity, human dECM‐based models may lack sufficient architectural guidance for proper tissue organization, potentially due to limited matrix remodeling or alterations introduced during decellularization [31, 33]. Moreover, SCs within human‐derived organoids, typically using Matrigel, frequently display phenotypic instability over time, with evidence of dedifferentiation and loss of junctional organization, likely due to the absence of sustained niche cues and insufficient matrix remodeling capacity. As a result, the establishment and maintenance of a functional BTB are compromised, limiting the organoids capacity to support long‐term SC functionality [21, 29, 34]. By contrast, the CTE offers a structurally coherent and biologically active environment, endogenously enriched in key ECM components such as laminin, fibronectin, and collagen IV, which are progressively remodeled by SCs and contribute to their gradual maturation. The capacity of the CTE to integrate both biochemical and biomechanical specificity enables not only SC viability but also their spatial reorganization, tight junction assembly, and upregulation of functional markers, which are rarely achieved with synthetic or exogenous matrices. Additionally, the use of spheroid‐seeded constructs appears to further potentiate this effect, providing intrinsic spatial cues that guide self‐organization and recapitulate early morphogenetic processes typical of seminiferous tubule formation during testicular development [35].

## 5 Conclusion

This study provides evidence that a structurally and biochemically defined ECM microenvironment is sufficient to support the long‐term functionality of hSCs, which is a fundamental requirement for establishing a permissive somatic niche in vitro. The engineered CTE, composed of human fibroblast‐derived matrix enriched in key testicular ECM components, facilitated progressive hSC integration, expression of maturation markers (SOX9, ABP), and organization of tight junction proteins (ZO‐1, OCLDN), along with active ECM remodeling. Notably, the spheroid‐based model enhanced phenotypic stabilization and barrier formation, underscoring the role of spatial cell organization in promoting SC identity and functional specialization. These findings highlight how ECM composition and architecture jointly regulate hSC behavior through coordinated biochemical, structural, and mechanical cues, providing a more physiologically relevant alternative to models based on inert or non‐human matrices. While the present work focuses on establishing a stable and functional Sertoli cell niche, the CTE is intentionally designed as a foundational platform for future co‐culture studies. Although the present study demonstrates structural organization and ECM remodeling, the functional validation of spermatogenesis was not addressed and will be essential to establish in future investigations. By recapitulating the structural, biochemical, and mechanical hallmarks of the native testicular niche, our CTE platform bridges a critical gap in human reproductive biology, offering a robust foundation for fertility preservation, regenerative strategies, and in vitro spermatogenesis research.

## Conflicts of Interest

The authors declare no conflicts of interest.

## Supporting information




**Supporting File**: adhm71184‐sup‐0001‐SuppMat.docx.

## Data Availability

The data that support the findings of this study are available from the corresponding author upon reasonable request.

## References

[1] J. A. Grootegoed , M. Siep , and W. M. Baarends , “Molecular and Cellular Mechanisms in Spermatogenesis,” Best Practice & Research Clinical Endocrinology & Metabolism 14 (2000): 331–343, 10.1053/beem.2000.0083. 11097779

[2] M. K. Y. Siu and C. Y. Cheng , “Dynamic Cross‐Talk Between Cells and the Extracellular Matrix in the Testis,” BioEssays 26 (2004): 978–992, 10.1002/bies.20099. 15351968

[3] L. L. Richardson , H. K. Kleinman , and M. Dym , “Basement Membrane Gene Expression by Sertoli and Peritubular Myoid Cells In Vitro in the Rat,” Biology of Reproduction 52 (1995): 320–330. 7711202 10.1095/biolreprod52.2.320

[4] H. Wang , L. Yuan , J. Song , Q. Wang , and Y. Zhang , “Distribution of Extracellular Matrix Related Proteins in Normal and Cryptorchid Ziwuling Black Goat Testes,” Animal Reproduction 19 (2022): 20220005, 10.1590/1984-3143-AR2022-0005. PMC9170007 35712443

[5] J. P. Alves‐Lopes and J. B. Stukenborg , “Testicular Organoids: A New Model to Study the Testicular Microenvironment In Vitro?,” Human Reproduction Update 24 (2018): 176–191, 10.1093/humupd/dmx036. 29281008

[6] J. Zhang , J. Hatakeyama , K. Eto , and S. I. Abe , “Reconstruction of a Seminiferous Tubule‐Like Structure in a 3 Dimensional Culture System of Re‐Aggregated Mouse Neonatal Testicular Cells Within a Collagen Matrix,” General and Comparative Endocrinology 205 (2014): 121–132, 10.1016/j.ygcen.2014.03.030. 24717811

[7] K. Chui , A. Trivedi , C. Y. Cheng , et al., “Characterization and Functionality of Proliferative Human Sertoli Cells,” Cell Transplantation 20 (2011): 619–635, 10.3727/096368910X536563. 21054948 PMC4096632

[8] B. C. Prante , K. L. Garman , B. N. Sims , and J. S. Lindsey , “Matrix‐Coated Transwell‐Cultured TM4 Sertoli Cell Testosterone‐Regulated Gene Expression Mimics In Vivo Expression,” In Vitro Cellular & Developmental Biology—Animal 44 (2009): 434–443, 10.1007/s11626-008-9135-8. 18810563

[9] S. Sakib , A. Uchida , P. Valenzuela‐Leon , et al., “Formation of Organotypic Testicular Organoids in Microwell Culture,” Biology of Reproduction 100 (2019): 1648–1660, 10.1093/biolre/ioz053. 30927418 PMC7302515

[10] M. M. S. Reis , A. C. Moreira , M. Sousa , P. P. Mathur , P. F. Oliveira , and M. G. Alves , “Sertoli Cell as a Model in Male Reproductive Toxicology: Advantages and Disadvantages,” Journal of Applied Toxicology 35 (2015): 870–883, 10.1002/jat.3122. 25693974

[11] S. S. Pendergraft , H. Sadri‐Ardekani , A. Atala , and C. E. Bishop , “Three‐Dimensional Testicular Organoid: A Novel Tool for the Study of Human Spermatogenesis and Gonadotoxicity In Vitro,” Biology of Reproduction 96 (2017): 720–732, 10.1095/biolreprod.116.143446. 28339648

[12] F. Ibtisham , J. Wu , M. Xiao , et al., “Progress and Future Prospect of In Vitro Spermatogenesis,” Oncotarget 8 (2017): 66709–66727. 29029549 10.18632/oncotarget.19640 PMC5630449

[13] I. K. Cho and C. A. Easley , “Recent Developments in In Vitro Spermatogenesis and Future Directions,” Reproductive Medicine 4 (2023): 215–232, 10.3390/reprodmed4030020.

[14] M. A. H. Ley and M. Dym , “Immunocytochemistry of Extracellular Matrix in the Lamina Propria of the Rat Testis: Electron Microscopic Localization,” Biology of Reproduction 37 (1987): 1283–1289. 3327543 10.1095/biolreprod37.5.1283

[15] A. Sawaied , B. El Levy , E. Arazi , E. Lunenfeld , Q. Shi , and M. Huleihel , “Follicle‐Stimulating Hormone and Testosterone Play a Role in the Regulation of Sertoli Cell Functions Following Germ Cell Depletion In Vitro,” International Journal of Molecular Sciences 26 (2025): 2702, 10.3390/ijms26062702. 40141344 PMC11942298

[16] J. Yu , L. Hu , H. Li , et al., “Single‐Cell RNA Sequencing Uncovers Abnormal Sertoli‐Cell Elevation and Testicular Niche Impairment in the Transfemales's Testis,” Cell & Bioscience 15 (2025): 106, 10.1186/s13578-025-01445-3. 40685388 PMC12276673

[17] B. Durkut‐Kuzu and C. Celik‐Ozenci , “GDNF Enhances HGF‐Induced Tubulogenesis and Organization of Sertoli Cell,” Journal of Assisted Reproduction and Genetics 42 (2025): 2083–2098, 10.1007/s10815-025-03493-7. 40402398 PMC12229439

[18] M. E. Edmonds and T. K. Woodruff , “Testicular Organoid Formation is a Property of Immature Somatic Cells, Which Self‐Assemble and Exhibit Long‐Term Hormone‐Responsive Endocrine Function,” Biofabrication 12 (2020): 045002, 10.1088/1758-5090/ab9907. 32492667

[19] T. Yokonishi , T. Sato , K. Katagiri , M. Komeya , Y. Kubota , and T. Ogawa , “In Vitro Reconstruction of Mouse Seminiferous Tubules Supporting Germ Cell Differentiation1,” Biology of Reproduction 89 (2013): 1–6, 10.1095/biolreprod.113.108613. 23759307

[20] M. Kasravi , A. Ahmadi , A. Babajani , et al., “Immunogenicity of Decellularized Extracellular Matrix Scaffolds: A Bottleneck in Tissue Engineering and Regenerative Medicine,” Biomaterials Research 27 (2023): 00348, 10.1186/s40824-023-00348-z. PMC9912640 36759929

[21] M. Mincheva , R. Sandhowe‐Klaverkamp , J. Wistuba , et al., “Reassembly of Adult Human Testicular Cells: Can Testis Cord‐Like Structures be Created In Vitro?,” MHR: Basic Science of Reproductive Medicine 24 (2018): 55–63, 10.1093/molehr/gax063. 29294090

[22] C. Casale , G. Imparato , F. Urciuolo , and P. A. Netti , “Endogenous Human Skin Equivalent Promotes In Vitro Morphogenesis of Follicle‐Like Structures,” Biomaterials 101 (2016): 86–95, 10.1016/j.biomaterials.2016.05.047. 27267630

[23] C. Casale , G. Imparato , C. Mazio , P. A. Netti , and F. Urciuolo , “Geometrical Confinement Controls Cell, ECM and Vascular Network Alignment During the Morphogenesis of 3D Bioengineered Human Connective Tissues,” Acta Biomaterialia 131 (2021): 341–354, 10.1016/j.actbio.2021.06.022. 34144214

[24] A. Tanaka , M. Nagayoshi , S. Awata , I. Tanaka , and H. Kusunoki , “Differentiation of Human Round Spermatids Into Motile Spermatozoa Through In Vitro Coculture With Vero Cells,” Reproductive Medicine and Biology 8 (2009): 169–175, 10.1007/s12522-009-0030-0. 29699323 PMC5904850

[25] J. Tesarik , M. Guido , C. Mendoza , and E. Greco , “Human Spermatogenesis In Vitro: Respective Effects of Follicle‐Stimulating Hormone and Testosterone on Meiosis, Spermiogenesis, and Sertoli Cell Apoptosis,” The Journal of Clinical Endocrinology & Metabolism 83 (1998): 4467–4473, 10.1210/jcem.83.12.5304. 9851795

[26] L. I. Lipshultz , L. Murthy , and D. J. Tindall , “Characterization of Human Sertoli Cells In Vitro,” The Journal of Clinical Endocrinology & Metabolism 55 (1982): 228–237, 10.1210/jcem-55-2-228. 6123520

[27] M. E. Dasso , C. L. Centola , M. N. Galardo , M. F. Riera , and S. B. Meroni , “FSH Increases Lipid Droplet Content by Regulating the Expression of Genes Related to Lipid Storage in Rat Sertoli Cells,” Molecular and Cellular Endocrinology 595 (2025): 112403, 10.1016/j.mce.2024.112403. 39490730

[28] M. K. Skinnerf , S. M. Schlitz , and C. T. Anthony , “Regulation of Sertoli Cell Differentiated Function: Testicular Transferrin and Androgen‐Binding Protein Expression,” Endocrinology 124 (1989): 3015–3024. 2542008 10.1210/endo-124-6-3015

[29] G. Richer , Y. Baert , and E. Goossens , “In‐Vitro Spermatogenesis Through Testis Modelling: Toward the Generation of Testicular Organoids,” Andrology 8 (2020): 879–891, 10.1111/andr.12741. 31823507 PMC7496450

[30] A. Janecki , A. Jakubowiak , A. Steinberger , and A. Steinberger , “Regulation of Transepithelial Electrical Resistance in Two‐Compartment Sertoli Cell Cultures: In Vitro Model of the Blood‐Testis Barrier,” Endocrinology 129 (1991): 1489–1496. 1908377 10.1210/endo-129-3-1489

[31] B. de O Horvath‐Pereira , G. H. D. R. Almeida , L. N. da Silva Júnior , et al., “Biomaterials for Testicular Bioengineering: How Far Have We Come and Where Do We Have to Go?,” Frontiers in Endocrinology 14 (2023): 1085872, 10.3389/fendo.2023.1085872. 37008920 PMC10060902

[32] D. F. Duarte Campos , C. D. Lindsay , J. G. Roth , et al., “Bioprinting Cell‐ and Spheroid‐Laden Protein‐Engineered Hydrogels as Tissue‐on‐Chip Platforms,” Frontiers in Bioengineering and Biotechnology 8 (2020): 00374, 10.3389/fbioe.2020.00374. PMC7198818 32411691

[33] M. Hamzeh and M. Movahedin , “Appropriate Biomaterials for Scaffold Fabrication to Induce In Vitro Spermatogenesis,” Regenerative Biomedicine 1 (2025): 78–84.

[34] S. Sakib , A. Voigt , T. Goldsmith , and I. Dobrinski , “Three‐Dimensional Testicular Organoids as Novel In Vitro Models of Testicular Biology and Toxicology,” Environmental Epigenetics 5 (2019): dvz011, 10.1093/eep/dvz011. 31463083 PMC6705190

[35] D. Rebourcet , P. J. O'Shaughnessy , J.‐L. Pitetti , et al., “Sertoli Cells Control Peritubular Myoid Cell Fate and Support Adult Leydig Cell Development in the Prepubertal Testis,” Development 141 (2014): 2139–2149, 10.1242/dev.107029. 24803659 PMC4011090

