# Supplementary material for: Tuning the Testicular Microenvironment for Enhancing Human Sertoli Cells Maturation and Functionality In Vitro
Source: Adv Healthc Mater. 2026 Apr 24;15(22):e05848. doi: 10.1002/adhm.202505848 (PMC13279868; doi:10.1002/adhm.202505848)
Supplement: Supplementary file 1 — Supporting File: adhm71184‐sup‐0001‐SuppMat.docx. [file ADHM-15-0-s001.docx]

**SUPPLEMENTARY INFORMATION**

**S1 Picrosirius red analysis on CTE**

CTE were collected at day 0, fixed in 10% neutral buffered formalin, dehydrated, embedded in paraffin, and sectioned at 5 μm thickness, as reported in section 2.10/2.11. Sections were stained with Picrosirius Re) following manufacturer’s instructions to visualize collagen fibers. Stained slides were imaged under polarized light microscopy with Olympus BX53 light microscope (10x, 20x objective lenses) to assess collagen organization. The images highlight a well-defined fibrillar network with varying degrees of fiber thickness and alignment. These results provide a baseline assessment of collagen structure prior to cell-mediated remodeling during culture.

**~~
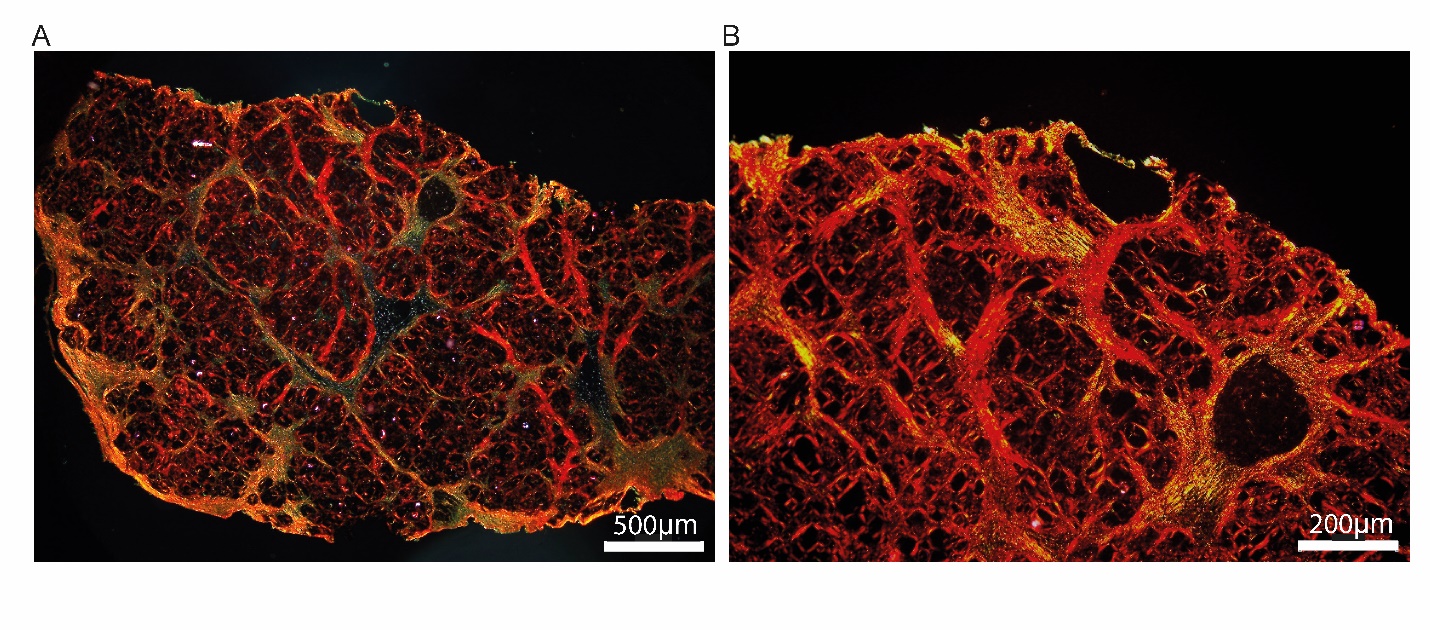
~~Figure s1: Picrosirius red analysis on CTE atDIV00, before hSC and hSC-sph seeding.**

**S.2 hSC-CTE characterization: immunofluorescence and histological analysis**

hSC were seeded onto CTEs, as reported in section 2.3. At DIV3 samples were fixed in 4% PFA for 30 minutes at RT, followed by PBS washes. Immunofluorescence analysis and imaging was performed as reported in section 2.8. The primary antibodies used were SOX9 (cyan), α-SMA (green, mouse polyclonal antibody, Abcam), ZO-1 (cyan) and OCLDN (cyan). As a secondary antibody, Alexa Fluor 488-conjugated goat anti-rabbit IgG (H + L) and Alexa Fluor 555-conjugated goat anti-mouse IgG (H+L) secondary antibodies (1:500, Invitrogen, Italy). Samples were observed using a Confocal Leica TCS SP5 II coupled with a Multiphoton Microscope (Leica Microsystems, Italy). Immunofluorescence imaging at day 3 confirmed marker expression in CTE cultured with HSC or HSC spheroids. Cells exhibited positive staining for α-SMA, indicating acquisition or maintenance of a contractile phenotype, and SOX9, associated with matrix remodeling or lineage-specific activity (**Figure s2A**). Although ZO-1 and OCLDN are detectable at DIV 3, their spatial distribution is still diffuse, and clear junctional organization cannot yet be identified. This suggests that tight junction assembly may not be fully established at this stage.

**
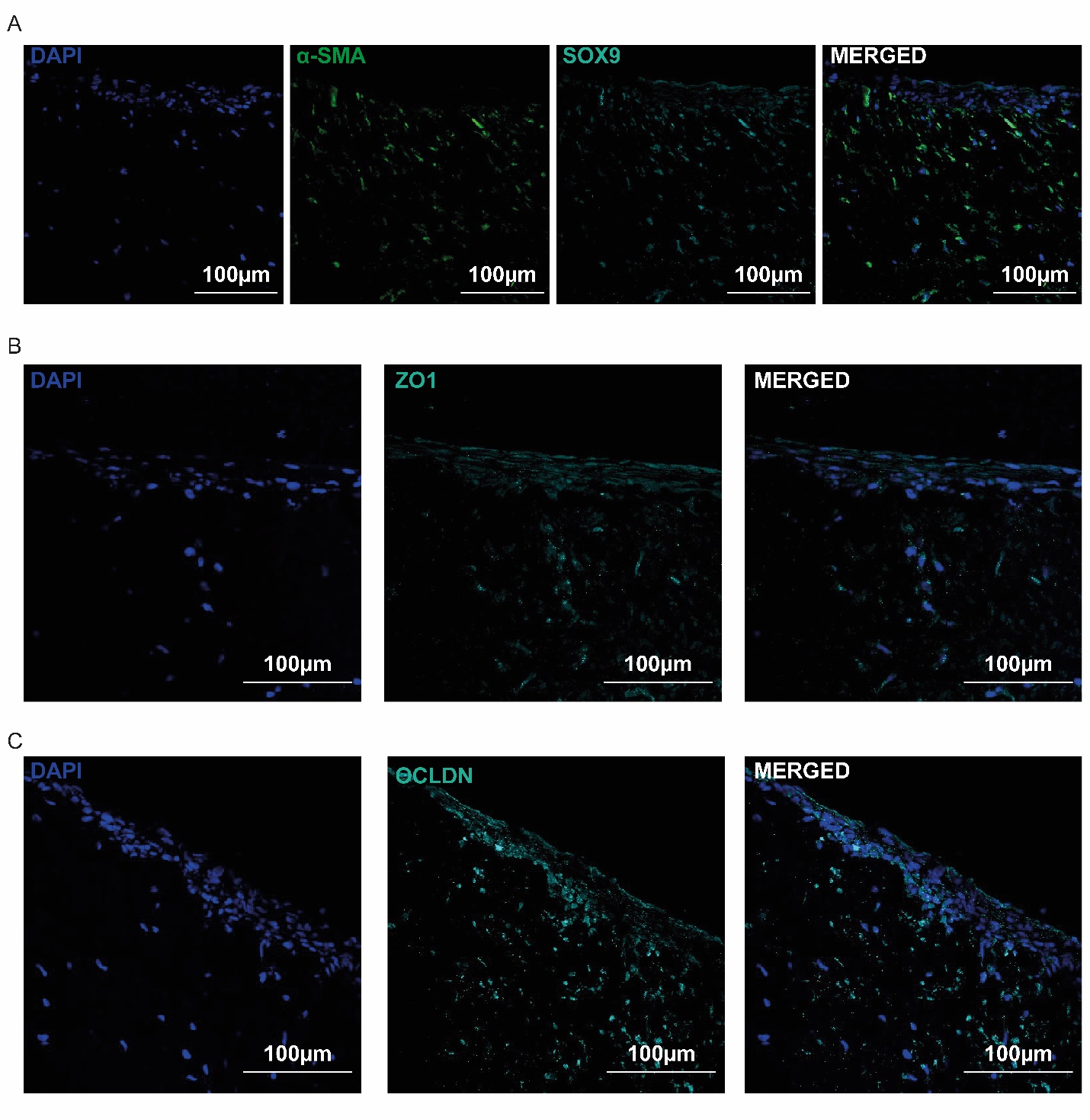
**

Figure s2: Immunofluorescence staining of CTE at day 3 cultured with hSC or hSC- sph. (A) α-SMA and SOX9 expression. (B) ZO-1 expression. (C) Occludin (OCLDN) expression. Nuclei were counterstained with DAPI.

**~~S.1 Materials and methods~~**

**~~S.1.5 hSC-CTE characterization: immunofluorescence and histological analysis~~**

~~hSC were seeded onto CTEs, as reported in section 2.3. At DIV7 and DIV14, samples were fixed in 4% PFA for 30 minutes at RT, followed by PBS washes. Immunofluorescence analysis and imaging was performed as reported in section 2.4. The primary antibodies used were SOX9 (cyan), α-SMA (green, mouse polyclonal antibody, Abcam), ZO-1 (cyan) and OCLDN (cyan). As a secondary antibody, Alexa Fluor 488-conjugated goat anti-rabbit IgG (H + L) and Alexa Fluor 555-conjugated goat anti-mouse IgG (H+L) secondary antibodies (1:500, Invitrogen, Italy). To evaluate collagen production and organization, samples were analyzed using a Confocal Leica TCS SP5 II coupled with a Multiphoton Microscope (Leica Microsystems, Italy). The imaging system was equipped with a near-infrared femtosecond laser, generated by a tunable mode-locked titanium:sapphire laser (Chameleon Compact OPO-Vis, Coherent). Second Harmonic Generation (SHG) imaging was employed to visualize collagen, utilizing an excitation wavelength (λEX) of 840 nm and detecting emission at 420 ± 5 nm.~~

~~The area occupied by SOX9 and α-SMA markers was analyzed using ImageJ software. The fluorescence signal area per cell was normalized to the total number of DAPI-stained nuclei. Data were represented as mean ± standard deviation (SD) and statistically analyzed using Student’s t-test to assess significant differences (**p < 0.0001, ******).~~

~~Histological analyses were performed on paraffin-embedded hSC-CTE samples at DIV7 and DIV14, following protocols in sections 2.6 and 2.7.~~

**~~S.2 Results~~**

**~~S.2.1 Optimization of hSC Culture Conditions in a 2D Transwell System~~**

~~To optimize culture conditions for hSCs in a 2D Transwell system, we evaluated their morphology, barrier properties, and ultrastructural features under different conditions. SEM images revealed that hSCs adhered to the fibronectin-coated Transwell membrane, exhibiting an elongated morphology with extensive cytoplasmic extensions. Barrier integrity was assessed via TEER measurements at different time points (DIV 1,4,7). A significant increase in TEER values was observed in hSCs cultured with FSH and Testosterone (hSC-FSH-T), with an even greater enhancement under ALI conditions (hSC-FSH-T-ALI) (p < 0.0001), indicating improved barrier function in response to hormonal stimulation and ALI exposure. These results indicate that hormonal stimulation and ALI exposure enhance the barrier function of hSC monolayers. TEM further confirmed these findings, showing the presence of well-formed tight junctions (white arrows), which are crucial for maintaining the blood-testis barrier. Additionally, lipid droplets (yellow) were observed in the cytoplasm, suggesting active metabolic activity and lipid storage, which are characteristic features of mature Sertoli cells. The presence of extracellular matrix (ECM, red) and distinct irregularly shaped nuclei (N, blue) further indicated a well-organized cellular architecture.~~

**~~Figure S1:~~** ~~Optimization of hSC Culture Conditions in 2D Transwell System. (~~**~~A~~**~~) TEER measurements over time, comparing different culture conditions: hSC (control), hSC-T-FSH (treated with testosterone and FSH), and hSC-T-FSH-ALI (treated with testosterone and FSH under ALI conditions). Statistics: ****p < 0.0001, *p < 0.001. (~~**~~B~~**~~) SEM images of hSC-T-FSH-ALI cultured in 2D Transwell inserts, showing cellular morphology and interaction with the substrate. Scale bars: 10 µm. (~~**~~C~~**~~) TEM images of hSCs-T-FSH-ALI, highlighting extracellular matrix (ECM, red), tight junctions (TJ, white arrows), nuclei (N, blue), and lipid droplets (LD, yellow). Scale bars: 2 µm (left image), 1 µm (central and right image).~~

**~~S.2.2 Characterization of hSC in CTE Constructs~~**

~~To investigate hSC differentiation and structural organization in CTE constructs, we analyzed samples at DIV7 and DIV14. Immunofluorescence staining revealed a significant increase in SOX9+ cells at DIV14 compared to DIV7 (p < 0.0001), suggesting active proliferation of hSC within the construct. In contrast, the population of α-SMA+ cells remained stable over time, suggesting that these cells did not undergo significant expansion. The quantitative analysis further confirmed these observations, showing that the progressive increase in SOX9+ cells at DIV14 suggests that the culture conditions support Sertoli cell proliferation while maintaining their identity. The stable α-SMA+ cell population indicates that these cells, likely fibroblasts with a myofibroblastic phenotype, do not expand over time. This phenotype is consistent with peritubular myoid cells of the testis, which are known to express α-SMA and contribute to the testicular microenvironment. FIB-SEM imaging at DIV14 revealed the formation of stratified cellular layers, indicating progressive cellular integration within the CTE matrix. These results demonstrate that hormonal stimulation and ALI culture conditions promote Sertoli cell expansion while maintaining their phenotypic identity.~~

**Figure S2~~:~~** ~~Characterization of hSC in CTE at DIV7 and DIV14. (~~**~~A~~**~~) Immunofluorescence staining of SOX9 and α-SMA in CTE-seeded cells at DIV7 (i) and DIV14 (ii). DAPI (blue) stains cell nuclei, SOX9 (cyan) marks human Sertoli cells (hSC), and α-SMA (green) identifies myofibroblasts. Merged images show the spatial distribution of both cell types within the connective tissue equivalent (CTE). Scale bars: 100 µm. (~~**~~B~~**~~) FIB-SEM images at DIV14, showing stratified cellular layers above the CTE structure. Scale bars: 5 µm. (~~**~~C~~**~~) Quantification of SOX9+ and α-SMA+ cells at DIV7 and DIV14. Statistics: ****p<0.0001.~~

~~Histological analysis at DIV7 and DIV14 confirmed these findings. H&E staining at DIV7 revealed a well-distributed cellular layer on the construct surface, while MT staining highlighted early ECM deposition, particularly collagen fibers. To assess the formation of tight junctions and barrier integrity, ZO-1 and OCLDN immunostaining were performed at both time points. At DIV7, ZO-1 (cyan) was localized at cell-cell junctions, indicating the establishment of early tight junctions. Similarly, OCLDN (cyan) immunostaining, together with SHG imaging, revealed initial fibrillar collagen structures at this stage. By DIV14, a more organized ZO-1 network was observed, suggesting an enhancement of junctional integrity over time. OCLDN expression was also increased, and SHG imaging showed a denser collagen network, indicative of progressive ECM remodeling and tissue maturation. Given that these properties were more developed at DIV14, this time point was chosen as the starting condition for spheroid-based model analyses.~~

**~~Figure S3:~~** ~~Histological and Immunofluorescence Analysis of CTE-seeded Cells at DIV7 and DIV14. (~~**~~A~~**~~) Histological staining at DIV7. (i) H&E staining showing cellular distribution and tissue organization. (ii) MT staining highlights ECM deposition and collagen fibers. Scale bars: 200 µm (left image) and 100 µm (right image). (~~**~~B~~**~~) Immunofluorescence staining at DIV7. (i) ZO-1 (cyan), a tight junction marker, is localized at cell-cell junctions, with DAPI (blue) marking nuclei. (ii) OCLDN (cyan) and SHG imaging visualize tight junctions and fibrillar collagen structures, respectively. Merged images highlight their spatial organization. (~~**~~C~~**~~) Immunofluorescence staining at DIV14. (i) ZO-1 (cyan) localization in cells stratified above the CTE, with DAPI (blue) staining nuclei. (ii) OCLDN (cyan) and SHG imaging, showing enhanced junction formation and collagen organization at later time points. Scale bars: 100 µm.~~
